# Patient-level simulation models in cancer care: a systematic review

**DOI:** 10.3389/fpubh.2025.1335300

**Published:** 2025-05-09

**Authors:** Sara-Lise Busschaert, Helena Van Deynse, Mark De Ridder, Koen Putman

**Affiliations:** Department of Public Health, Vrije Universiteit Brussel, Brussels, Belgium

**Keywords:** discrete event simulation, microsimulation, cancer, health economics, health policy and management

## Abstract

**Background:**

Patient-level simulation (PLS) models overcome some major limitations of conventional cohort models and have broad applicability in healthcare, yet limited knowledge exists about their potential in cancer care.

**Objectives:**

This systematic review aims to: (1) describe the application areas of PLS models in cancer care, (2) identify commonly used model structures, (3) evaluate the quality of reporting based on established guidelines, and (4) critically discuss the potential and limitations of PLS models in this context.

**Methods:**

A systematic literature search was completed in Web of Science, PubMed, EMBASE and EconLit. Reasons underlying the use of PLS models were identified with a conventional inductive content analysis and reporting quality was assessed with an 18-item checklist based on the ISPOR-SMDM guidelines.

**Results:**

The number of publications increased over time and most studies used state-transition microsimulation (49.25%) or discrete event simulation (48.51%). Two main application areas could be discerned, namely disease progression modelling (DPM) (78.36%) and health and care systems operation (HCSO) (21.64%). In the DPM domain, the use of PLS models was mainly motivated by the need to represent patient heterogeneity and history. In the HCSO domain, PLS models were used to better understand and improve cancer care delivery. Average reporting quality was 65.2% and did not improve over time.

**Conclusion:**

PLS models can be used to simulate the progression of cancer and to model cancer care delivery. In the DPM domain more direct comparisons with cohort models are required to establish the relative advantages of PLS models and in the HCSO domain the impact on clinical practice needs to be systematically assessed. Furthermore, adherence to the ISPOR-SMDM guidelines should be improved.

## Introduction

Cancer is a critical global health challenge, with close to 20 million new cases and 9.7 million cancer-related deaths reported globally in 2022 alone (1). According to updated estimates from the International Agency for Research on Cancer, approximately one in five individuals will develop cancer in their lifetime, and around one in nine men and one in 12 women will die from it. With predictions that new cancer cases may rise to 35 million annually by 2050, the importance of preventive measures, early detection, and equitable access to care is more urgent than ever.

Cancer incidence tends to increase with a country’s Human Development Index (1), and in Europe and North America, the disease has become the leading cause of death among middle-aged adults (2). However, in low- and middle-income countries (LMICs), the cancer burden is also rapidly increasing, with cancer rates rising as these nations undergo demographic and epidemiological transitions. Survival rates are lower in LMICs due to limited access to screening, early diagnosis, and effective treatment, and global efforts to address these disparities are therefore critical for improving cancer outcomes across all regions.

To encourage progress against cancer the pace of innovation in oncology has accelerated at an unprecedented speed in recent years, yet novel treatments come with an increasing price tag (3, 4). In a world where resources are finite and healthcare systems are under increasing strain, the question of how to improve value in cancer care has never been more pertinent. In this context, “value” is defined as the ratio of patient outcomes to the cost of care (5–7). The goal is to achieve the best possible outcomes for patients while ensuring that the cost of treatments, remains sustainable for healthcare systems and affordable for patients. The rising costs associated with cutting-edge cancer treatments, coupled with the need to ensure access to quality care for all patients, have made value-based healthcare models a focal point of policy discussions and health economic research.

Achieving high-value cancer care requires an evidence-based approach to assess the effectiveness and cost-effectiveness of treatments. Decision analytic models address this need and provide a framework to integrate the best available evidence on the decision problems encountered within healthcare (8, 9). Models must, however, adequately represent the complexities of clinical reality to be a valid foundation on which to base decisions (10, 11). With respect to cancer care, this is a particularly challenging endeavor (12). Treatment for cancer generally involves a combination of therapies (13), delivered simultaneously or sequentially, and requires coordination between multiple disciplines (14). Moreover, in the fast-paced field of oncology, more sophisticated therapies emerge continuously, and treatment paradigms are rapidly evolving. In this dynamic new era, cancer is gradually changing from a death sentence to a chronic disease that requires long-term management (15) and treatments are becoming increasingly personalized (16, 17) in recognition of the substantial heterogeneity that underlies the disease (18).

The growing complexity of cancer care may pose challenges for Markov and other cohort models, the dominant modelling approach today (19, 20). In a cohort model outcomes are calculated for a cohort of supposedly average patients, without considering potential differences between patients. This traditional modelling technique is subject to a number of restrictive assumptions, which may limit its capacity to accurately represent the nature of cancer and the realities of cancer care delivery (12). Markov models presume that the population of interest is homogenous, and may therefore struggle to adequately reflect the heterogeneity in cancer patients (21). Furthermore, the Markovian memoryless property and the frequent use of discrete time cycles could hamper a proper consideration of the dynamics of cancer (22–24).

For these reasons, it has been suggested that researchers should turn to alternative methods such as patient-level simulation (PLS) models (25, 26). As defined by Drummond et al. (9), these models calculate the outcomes of a sufficiently large sample of simulated patients and subsequently average across patients. Since PLS models provide estimates for the outcomes of interest for each individual patient they allow modelers to examine variability in outcomes and to track individual patient histories (21). Moreover, PLS models are not constrained by the Markov property and may therefore be better able to accurately reflect the dynamics of cancer (21, 27). Errors arising from the use of discrete time cycles could be averted as well, either by opting for continuous time state-transition microsimulation (22) or for discrete event simulation (DES) (25).

Importantly, the application of PLS models is not limited to the area of health economics and health technology. In the field of health policy, the approach is used to simulate health trajectories and to predict the impact of alternative policy interventions (28). Notable examples include the Future Elderly Model (FEM) (29) and the Population Health Model (POHEM) (30), two continuous time state-transition microsimulation models. Discrete event simulation, on the other hand, is increasingly applied in healthcare management to analyze how resources can be optimally employed (31). The apparent versatility of PLS models thus suggests they may be of use to researchers in a variety of healthcare fields (25).

While PLS models have demonstrated their utility in various healthcare domains, their application to cancer care remains underexplored. To our knowledge, no systematic review has examined the application of PLS models in cancer care (19). This knowledge gap may reflect both the technical challenges of implementing PLS models and the historical dominance of cohort-based approaches. Nevertheless, as cancer care continues to evolve, the time is ripe to investigate the potential of PLS models to enhance decision-making in oncology.

In this systematic review, we aim to address this gap by laying the groundwork for future research and providing a roadmap for integrating PLS models into oncology. We document the utilization of PLS models across time, examine which specific techniques are favored and which questions they are applied to. Moreover, we systematically assess the reporting quality of papers and conduct a content analysis to uncover the main reasons for choosing a PLS model. Finally, we critically discuss the potential contribution of PLS models in cancer care.

## Methods

### Search strategy

A systematic search of the peer-reviewed literature was conducted in four major databases, namely, Web of Science, PubMed, EMBASE and EconLit. The search strategy was first developed for Web of Science, and subsequently adapted for the remaining databases. Search terms were identified through consultation of guidelines (32), textbooks (9) and earlier reviews (19, 26, 33), as well as through exploration of databases. The full search strategy for each database can be consulted in Supplementary Appendix 1. Since PLS models were relatively rare before 2010 (26, 33), January 2010 was chosen as the start of the time frame.

### Inclusion and exclusion criteria

To be eligible for inclusion publications needed to include an application of the PLS approach to cancer treatment. In addition, models needed to be clearly described to permit a classification of modelling techniques according to the International Society for Pharmacoeconomics and Outcomes Research–Society for Medical Decision Making (ISPOR-SMDM) Modelling Good Research Practices Task Force guidelines (11, 32). Models also needed to be fully developed and applied to a certain research question to enable us to explore the application areas of the PLS approach. Gray literature was excluded to maintain a focus on peer-reviewed publications. The inclusion and exclusion criteria are summarized in Table 1.

**Table 1:** Inclusion and exclusion criteria.

Domain	Inclusion criteria	Exclusion criteria
Population	Cancer patients	Patients with other diseases, or people at increased risk of cancer (e.g., smokers).
Intervention	All types of cancer treatment (i.e., radiotherapy, chemotherapy, hormone therapy, surgery, immunotherapy, targeted therapy, active surveillance, other).	Cancer prevention, cancer screening.
Model type	All types of PLS model (i.e., state-transition microsimulation, discrete event simulation, agent-based model, partially observable markov decision process, other).	Cohort models, biological or animal models of cancer.
Model description	Article must include a clear description of the PLS model.	Articles that only refer to a PLS model without further explanation.
Model development	The PLS model must be fully developed and provide answers to a clearly defined research question.	Models that are still a work-in-progress, that do not provide a research question and/or results.
Language	English, Dutch, German, French	Other languages
Publication type	Research articles	Conference abstracts, reviews, editorials and expert opinions.

### Data extraction

Titles and abstracts of initially retrieved studies were screened independently by two reviewers using the web application Rayyan. Selection was based on the predefined eligibility criteria and the two reviewers (SLB and HVD) were blinded to each other’s decision. In cases of disagreement, consensus was reached through discussion, and if necessary, a third reviewer (KP) was consulted. Subsequently, the full texts of eligible articles were obtained and screened according to the same criteria. One reviewer (SLB) assessed all full-text articles, and a second reviewer independently checked a random sample of 20%, which is judged to be representative (19). Agreement rate was 100% and inter-rater reliability was therefore deemed sufficiently high. Key data, including study objectives, modelling techniques, application areas, and results, were extracted using a standardized data extraction form developed based on PRISMA guidelines.

To gain insight into the primary reasons underlying the use of PLS models a conventional inductive content analysis was conducted according to Elo and Kyngäs (34). This rigorous and thorough method for analysing text-based data is well-suited for identifying major themes and objectives and is recommended when there is little existing research on a topic (35, 36). The approach involved open coding of the text by two reviewers (SLB and KP), who iteratively developed and refined categories to capture emerging themes. The analysis was performed using Nvivo, which is well-suited for qualitative analysis of text-based data.

Reporting quality was assessed with an 18-item checklist for PLS models based on the ISPOR-SMDM guidelines (37). The checklist was designed to be applicable to model-based analyses in a variety of healthcare fields, including but not limited to health economics and healthcare management. Scoring was conducted independently by two reviewers (SLB and KP), and discrepancies were resolved through consensus. A two-way ANOVA was performed that examined the effect of application area and modelling technique on reporting quality. Prior to analysis, the assumptions of ANOVA were tested, including normality of residuals (using the Shapiro–Wilk test) and homogeneity of variances (using Levene’s test). Results were analyzed with IBM SPSS Statistics 28.0.

## Results

### Search results

**Figure 1:**
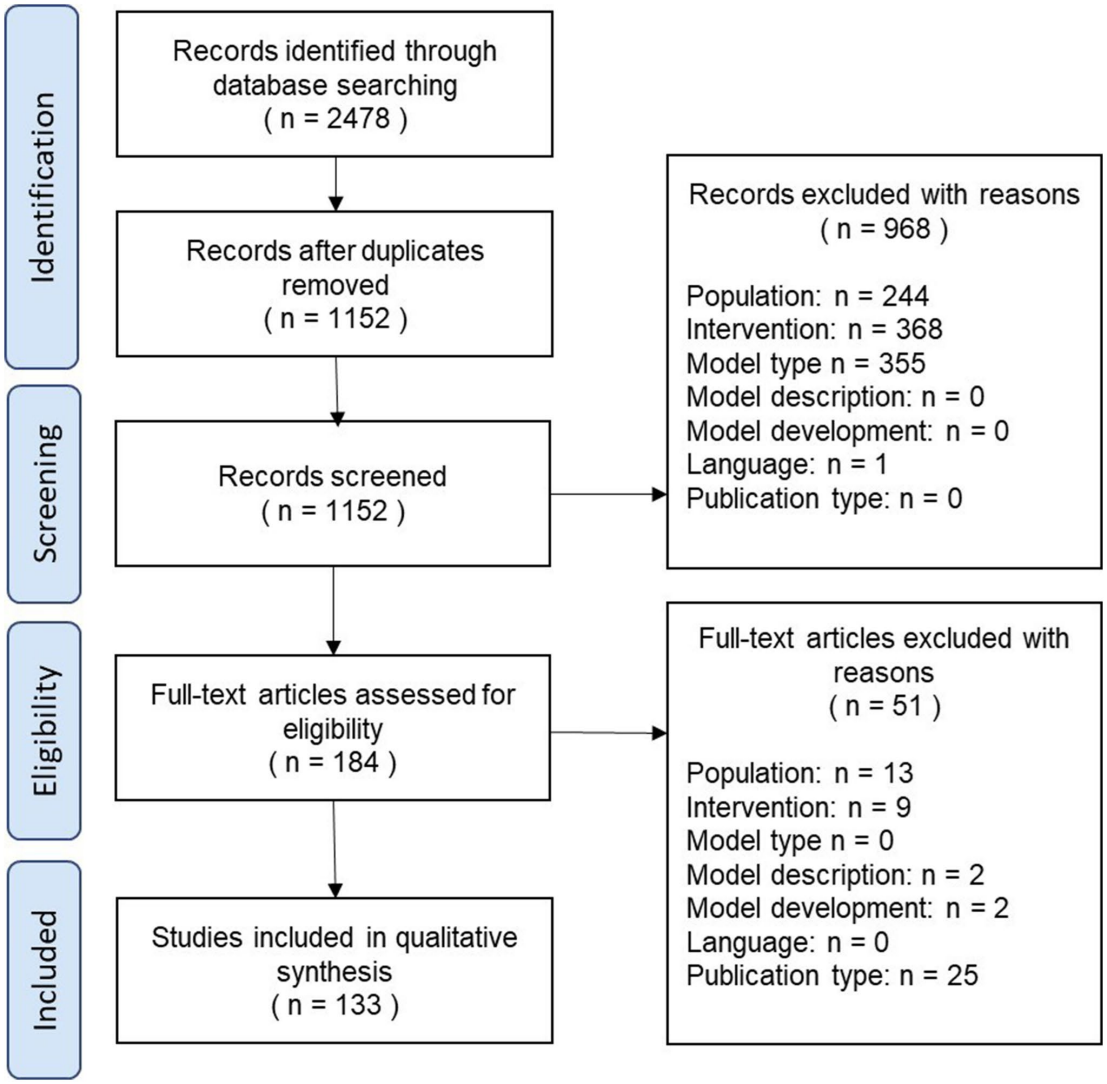
PRISMA diagram.

The results of the review process are illustrated as a PRISMA diagram in Figure 1.

### Publication trends over time

**Figure 2:**
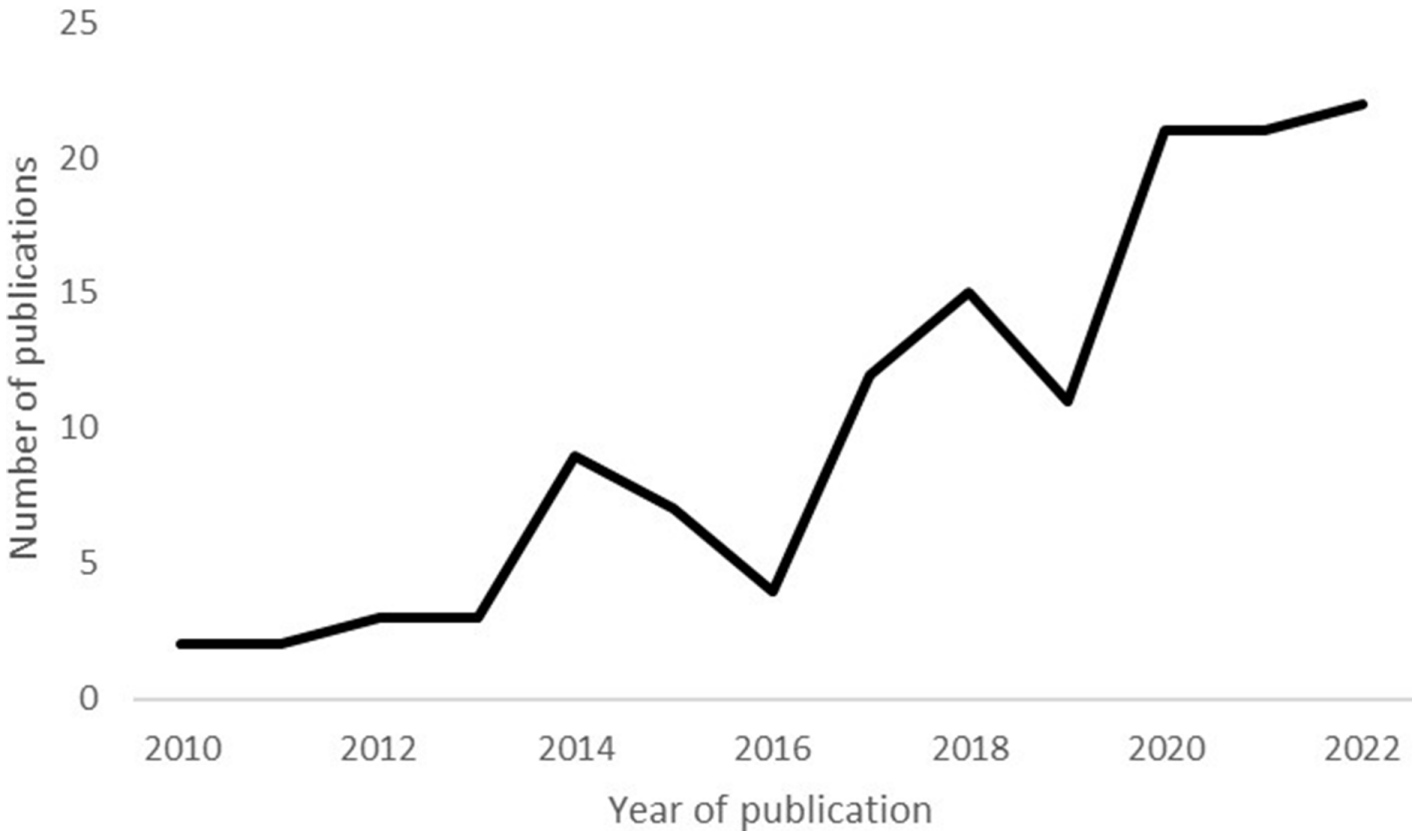
Number of publications per year.

Figure 2 illustrates a clear increase in the number of published PLS models in recent years. Only eight papers were published between 2010 and 2012, but this number increased eightfold between 2019 and 2021. These results are consistent with earlier reviews which documented an increasing utilization of PLS models (26, 33).

### Model structures

The majority of studies used either state-transition microsimulation (STMS) (49.25%) or discrete event simulation (DES) (48.51%). Other model structures (2.24%) were relatively rare and included agent-based modelling (*n* = 1) and timed automata (*n* = 1).

### Application areas

To investigate the areas in which the PLS approach has been applied, the models were categorized according to a classification scheme inspired by an earlier review (19). Broadly, the models in the present review can be grouped into two major categories, namely disease progression modelling (DPM) and health and care systems operation (HCSO). In what follows, we give an overview of the general features of these models. A summary of the results for DPM and HCSO models can be found in Tables 2, 3, respectively. More detailed information is provided. More detailed information is provided in Supplementary Appendices A1, A2.

**Table 2:** Characteristics of DPM models.

Domain	Number	Percentage
Model type		
State-transition microsimulation	65	61.90
Discrete event simulation	39	37.14
Timed automata	1	0.95
Subcategories		
Cost-effectiveness	74	71.15
Health outcomes	25	24.04
Health outcomes and costs	3	2.88
Costs	2	1.92
Patients (cancer type)		
Breast	17	16.19
Prostate	15	14.29
Lung	12	11.43
Colorectal	8	7.62
Other	52	50.00
Study setting (World Bank classification)		
High income countries	88	83.81
Low and middle income countries	5	4.76
Unclassified*	12	11.43

*Papers that did not report patients’ nationality or that included data from patients from both high as low and middle income countries are categorized as unclassified.

**Table 3:** Characteristics of HCSO models.

Domain	Number	Percentage
Model type		
DES	26	89.66
Microsimulation	2	6.90
Agent-based modelling	1	3.45
Subcategories		
Patient scheduling	10	34.48
Operational changes	9	31.03
Capacity planning and management	5	17.24
Resource allocation	2	6.90
Miscellaneous	2	6.90
Resource scheduling	1	3.45
Outcomes*^1,2^		
Waiting time	19	65.52
Throughput time	8	27.59
Resource utilization	5	17.24
Throughput	5	17.24
Working time	4	13.79
Overtime	4	13.79
Cost	2	6.90
Resource utilization	2	6.90
Health outcomes	1	3.45
Simulated setting		
Micro-system	27	93.10
Macro-system	2	6.90
Patients (cancer type)		
Not mentioned	17	62.07
Multiple	3	10.34
Lung	2	6.90
Skin	2	6.90
Breast	1	3.45
Colorectal	1	3.45
Bladder	1	3.45
Pediatric	1	3.45
Study setting (World Bank classification)		
High income countries	25	86.21
Low and middle income countries	4	13.79

*1The total number of measured outcomes is greater than the number of HCSO publications because several papers assessed multiple outcomes.

*2Throughput refers to the number of patients that pass through the modeled system during a given time period and throughput time denotes the amount of time it takes for a patient to pass through the modeled system.

#### Disease progression modelling

Disease progression models, which comprise the largest category (78.36%), describe the time course of diseases and simulate the influence of treatment options on disease status (38–40). Accordingly, these models provide guidance in medical decision-making by indicating the best course of action based on clinical benefits and harms, consumed resources or both. Within this area of application, STMS was the most commonly employed technique (61.90%). DPM models can be organized into subcategories according to the outcomes that are used to compare alternative treatment strategies. The majority of DPM models evaluated therapeutic interventions for cancer based on their cost-effectiveness (71.15%), expressed as an incremental cost-effectiveness ratio (ICER). The remainder considered only health outcomes (e.g., QALYs), only costs (e.g., healthcare costs) or both costs as health outcomes without combining them in a cost-effectiveness analysis. With regard to the patient populations, the four cancer types with the highest incidence worldwide (i.e., breast, prostate, lung, and colorectal cancer) were predominantly studied (41). Furthermore, patient populations generally came from high-income countries, which constitute the regions with the highest cancer incidence (41).

Notably, certain research questions were frequently addressed. To begin, several authors investigated the value of applications in the domain of personalized oncology (42–64), for example by assessing the cost-effectiveness of molecular diagnostics (47, 50, 60, 61), or by demonstrating that PLS models can be used to guide radiotherapy decisions (56, 59). The PLS approach was likewise often applied to model sequences of treatments and/or tests (42, 44, 51–55, 61, 64–78). Blommestein et al. (67), for instance, determined the cost-effectiveness of thirty treatment sequences including up to three lines of therapy for patients with multiple myeloma. A number of authors also used PLS models to represent the full clinical trajectory of a disease (79–81). An example is the model by Wang et al. (81), which simulates costs, survival and QALYs for patients with follicular lymphoma across the treatment pathway. Other models included not only treatments, but also elements such as screening and prevention (69, 82–89). Tappenden et al. (84), for instance, constructed a whole disease model of colorectal cancer, which proved capable of evaluating the majority of topics within the UK’s NICE colorectal cancer guideline within one consistent framework.

#### Health and care systems operation

HCSO models simulate healthcare systems and can be harnessed by healthcare managers to better understand and improve the delivery of care (90). The domain of HCSO was nearly monopolized by DES models (89.65%), and most researchers turned their attention to issues touching on patient scheduling and operational changes. To evaluate the effects of proposed alterations modelers typically focused on operational outcomes, particularly waiting time. The ABM model, as well as most DES models, were unit-specific, that is, they represented specific hospital departments dedicated to the delivery of cancer drugs, radiotherapy or surgery (i.e., micro-systems). The two papers on STMS, on the contrary, modeled the healthcare system at a national level (91, 92) (i.e., macro-systems).

We also examined the extent to which proposed quality improvements were implemented in clinical practice, given that it is assumed that the impact of HCSO models in healthcare is quite limited (93, 94). In this review, 27.59% of the authors did not explore any scenarios to improve the modeled system or explicitly stated that implementation was not their intention (91, 92, 95–99). In the remaining publications an implementation was mentioned in 38.10% of the cases (100–107) and 19.05% (100, 101, 105, 106) also reported an evaluation of the intervention.

### Reasons for using PLS models

The reasons for choosing a PLS model were broadly in line with the presumed advantages reported in the literature. Within the DPM category, model choice was frequently motivated by the presence of heterogeneity in patient characteristics and pathways (47, 50, 51, 53, 55, 61, 63, 65, 67, 74, 78–81, 108–118) or the importance of taking into account patient history (47, 50, 52, 68, 72, 79, 80, 108–110, 113, 115, 116, 119–122). Notably, a number of authors within the DPM domain also drew attention to specific advantages of the DES framework, such as the avoidance of errors due to the use of discrete time cycles (42, 51, 115, 119, 123) and a more efficient handling of competing risks (124). One paper also cited the ability to represent resources and to simulate the effects of wait times (125). All other DES models in the DPM category were, however, non-constrained resource models. Of note, a small number of DPM papers directly contrasted the PLS with the cohort approach. Jahn et al. (54) performed a cross-model validation of two models for personalized breast cancer treatment, a DES model and a cohort state-transition model, and observed that model choice can affect cost-effectiveness results. Gibson et al. (110) came to the same conclusion based on a comparison of the PLS approach with a survival partition and cohort model. Degeling et al. (115), in contrast, found comparable cost-effectiveness outcomes for a DES and a Markov model for metastatic colorectal cancer. Yet, the authors remarked that the DES model provided a more accurate representation of the clinical trial data in terms of mean health-state durations. In a similar vein, Pan et al. (116) reported that a DES model for prostate cancer predicted clinical outcomes from trial data more accurately than a survival partition model, especially over a longer time horizon.

HCSO modelers mainly used the PLS approach as a tool to gain knowledge about health care systems (91, 97–99, 101, 126–129) and to improve care delivery processes (101, 126, 130). In particular, PLS models can be used to explore a variety of “what if?” scenarios (100–103, 105, 106, 126, 127, 129–133) while avoiding the risks and costs that real-life experimentation would entail (101, 106, 130, 131, 133). Improvements that are cost-free or even cost-saving can be uncovered (101, 106, 130, 131, 133), and users can learn how to more efficiently employ scarce resources (134). Furthermore, the model can not only support informed decisions (103, 130), but can also serve as a communication tool to convince other stakeholder s (101, 133, 134).

### Reporting quality

**Figure 3:**
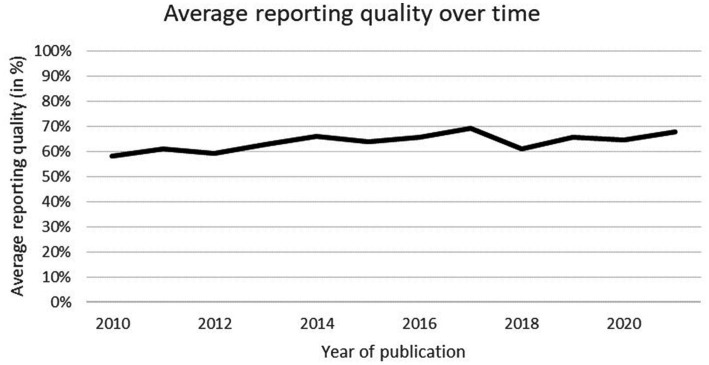
Average reporting quality (in %) per year.

The average reporting quality was 65.2% (SD = 11.4), and no significant time trend was observed, *R^2^* = 0.011, *F*(1, 132) = 1.427, *p* = 0.234 (see Figure 3). There was no significant interaction between the independent variables *F*(1,127) = 0.001, *p* = 0.981 and no significant difference between modelling techniques (*p* = 0.648). Nonetheless, simple main effects analysis did demonstrate that DPM models (x̄ = 68.59, SD = 8.07) obtained significantly higher scores than HCSO models (x̄ = 53.06, SD = 10.77) (*p* < 0.001).

**Figure 4:**
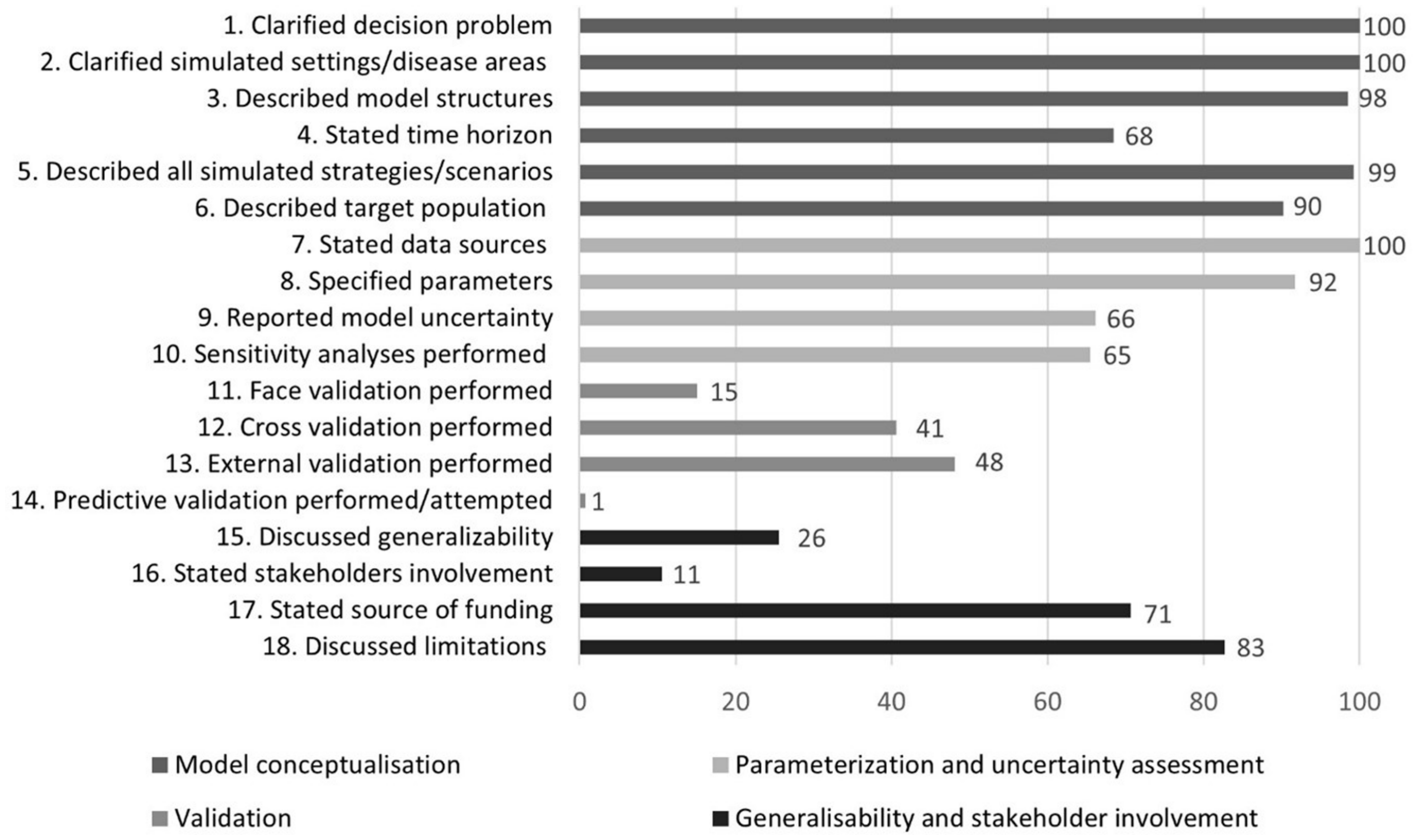
Fulfilment rates for individual items of the reporting quality checklist.

The fulfilment rate of individual checklist items is shown in Figure 4. Most criteria concerning model conceptualization (1–6) as well as those related to parameterization and uncertainty assessment (7–10) were fulfilled at high rates. Fulfilment rates for criteria regarding generalizability and stakeholder involvement (15–18), and especially those pertinent to validation (11–14), were, however, substantially lower.

## Discussion

We have documented an increasing use of PLS models in cancer care and identified two distinct application areas of PLS models, namely the simulation of the progression of cancer and the modelling of the healthcare systems in which patients are treated. Although DPM and HCSO models have different functions and intended users, they could both contribute to the achievement of high-value cancer care. To fully tap the potential of these models, however, some points warrant closer scrutiny (Table 4).

**Table 4:** Comparison of existing evidence and new insights from this review.

Domain	Existing evidence	New insights
Application of PLS models in cancer care	The potential of PLS models in oncology had not been extensively explored, and no systematic review had synthesized their use.	This review documented the increasing adoption of PLS models in cancer care and identified two primary applications: disease progression modelling (DPM) and healthcare system operations (HCSO).
Advantages of PLS over traditional cohort models in health economics	PLS models were hypothesized to offer advantages over cohort models, particularly by addressing key limitations such as the inability to capture individual variability and the constraints of the Markovian memoryless property.	Empirical evidence confirms that researchers frequently use PLS to model patient heterogeneity and history. PLS is often applied in contexts where these factors are critical, such as assessing personalized medicine interventions and modelling treatment sequences.
Use of PLS in healthcare system optimization	PLS models had been applied in healthcare operations research, but their effectiveness and real-world implementation had not been systematically assessed.	While PLS models are proposed as solutions for optimising healthcare delivery, their implementation in clinical practice remains limited, with fewer than 50% of studies reporting real-world application.
Reporting quality and validation practices	Previous research highlighted concerns regarding inadequate reporting and validation of cohort-based health economic models, but it was unclear whether these issues also applied to PLS models.	This study confirmed deficiencies in reporting, particularly concerning validation practices for PLS models, and emphasized the need for stricter adherence to reporting guidelines.
Future research directions	While there had been calls for broader adoption of PLS models in cancer care, a structured analysis of key research gaps was lacking.	This review identifies specific areas for improvement, including enhanced validation, direct comparisons with cohort models, and strategies to bridge the gap between model development and real-world implementation.

The table provides an overview of existing knowledge prior to this review and the novel insights generated through this systematic analysis.

### Choosing valuable treatments

DPM models, the most frequently used category in this review, primarily evaluated treatment cost-effectiveness. The decision to adopt the PLS approach was frequently motivated by the need to represent heterogeneity in patient characteristics and pathways or to model patient history. Accordingly, PLS models were often applied in areas where these aspects are likely to be of particular importance. A prime example can be found in personalized medicine, a burgeoning field of research in oncology (135). As it is challenging to accurately capture dynamic and diverse patient pathways in conventional cohort models, it has been argued that PLS models may be relatively superior in this area (26).

The modelling of sequences of treatments and/or tests is another area that may be well-suited to the PLS approach. Treatment sequences are a common feature of the contemporary management of metastatic cancer and identifying an optimal sequence for (subpopulations of) patients is the ambition of many researchers (136, 137). Answering such questions using randomized controlled trials is, however, unfeasible (65, 67) and the difficulty of efficiently representing downstream consequences of earlier treatments in cohort models could hinder an accurate assessment of the (cost) effectiveness of treatments across the entire sequence (138). The PLS approach likewise appears suitable for the construction of generic models of the full disease course of a certain type of cancer. Such comprehensive and adaptive models allow the comparison of multiple interventions across the whole cycle of care and permit nuanced judgments on which treatments to use for which patient subgroups (84, 139). These models go beyond standard technology adoption questions and could support the move toward a health technology assessment paradigm that supports health technology management, aims for efficiency and considers whole system value from a long-term perspective (140–142). This paradigm change is becoming more and more necessary due to the increasing strain on healthcare expenditures (143).

Although these results suggest that the PLS approach may have merit in a few noteworthy fields of research, the true litmus test for PLS models in the DPM domain is a head-to-head comparison with cohort models. In this review, four model comparisons were identified. This review’s observation that model choice can impact outcomes is particularly significant. In some cases, such as for the analysis of patient-level time-to-event data, a PLS model may be more appropriate than a cohort model (115, 116). Nevertheless, the scarcity of identified model comparisons precludes drawing conclusions about the relative merits of PLS models in cancer care and continuing work in this area therefore represents an important avenue for further research. Researchers should ideally also take into account potential drawbacks of the PLS approach, such as increased complexity, computational intensity and data requirements (8, 27). Although validity should always take precedence over simplicity, the ISPOR-SMDM Modeling Good Research Practices Task Force recommends that models should be kept as simple as possible (11). PLS models should therefore not replace cohort models as the default option in cancer care, but rather be employed when required by the demands of the decision problem (32).

### Improving the value of cancer care delivery

The HCSO domain formed the second important application area of PLS models in this review. Consistent with previous reviews (33, 93), models were generally unit-specific and simulated a certain clinic or a specific hospital department. In the HCSO domain, authors employed the PLS approach to acquire knowledge of health care systems and to improve care delivery processes. Nevertheless, although many papers in this review described solutions to enhance the quality of cancer care (95, 100, 101, 103, 107, 128, 130, 131, 144), it can currently not be established whether HCSO models lead to genuine benefits for patients or the organizations that care for them. Previous reviews have concluded that the application of modelling results in healthcare is remarkably low and it is estimated that 90% of these models have no influence on clinical practice (93, 145). In the present review, less than half of the papers that described experiments to improve modeled system mentioned an implementation in clinical practice and only about one in five also reported an evaluation of the intervention. It remains unclear whether these results stem from an implementation gap or inconsistent reporting. Promoting higher quality reporting, for example by encouraging the adherence to reporting guidelines such as those published by the World Health Organisation (146), could enable us to assess the scope of the implementation gap and may also reveal some of its causes. Bridging this gap is crucial, as research confined to academia does not directly benefit patients. Modelers may be advised to work out a systematic implementation strategy with key stakeholders to bridge the know-do gap. The Consolidated Framework for Implementation Research (CFIR) (147, 148), one of the most widely used implementation science frameworks, can assist researchers in planning for a successful implementation.

### Setting higher standards: enhancing reporting quality

The assessment of reporting quality in PLS models revealed notable deficiencies, particularly in the area of validation, underscoring the need for greater transparency and rigor in future research. The lack of detailed reporting on model validation—defined as the evaluation of whether a model is a proper and sufficient representation of the system for a particular application (149)—aligns with findings from previous reviews (37, 150). The importance of validation is paramount, as decision-analytic models are intended to guide decision-making. For these models to effectively serve that purpose, decision-makers must have confidence in the accuracy and reliability of the results.

Different types of validation play a crucial role in strengthening the credibility of decision-analytic models (151). Although 72% of the papers reviewed reported conducting one or more types of validation, significant gaps remain. Face validation, reported in only 15% of studies, ensures that the model aligns with current medical science and the best available evidence. Cross-validation, noted in 41% of studies, compares models using different methods to ensure similar outcomes. External validation, present in 48% of papers, confirms that model predictions align with real-world results, such as clinical trial outcomes. Predictive validation, regarded as the most esteemed form of validation due to its alignment with the core purpose of modelling—forecasting future outcomes—was reported in only 1% of studies.

Growing awareness of the significance of validation has prompted calls for adherence to standardized validation guidelines (152). Researchers employing PLS models should particularly heed this call to action, as these models tend to be more complex and less transparent, potentially undermining trust in their findings (27). Actively engaging with stakeholders can enhance the development of valid models, making them more relevant and practical (153). This collaborative approach could bridge the gap between academia and real-world healthcare, transforming theoretical exercises into actionable tools that provide tangible benefits.

## Limitations

This review is subject to some limitations that need to be mentioned. Firstly, the search was limited to peer-reviewed research articles, which may have led to the exclusion of some relevant papers. Nevertheless, our comprehensive search of four major databases likely provides a representative snapshot of the literature. Secondly, although agreement rate was 100%, the second reviewer only screened a representative sample of full-text papers.

## Conclusion

The cancer care landscape is rapidly evolving and new additions to the expanding therapeutic armamentarium arrive continuously, often accompanied with stratospheric costs and unclear benefits (154). Policymakers, healthcare managers and clinicians face difficult decisions under high uncertainty and require models that accurately reflect the complexities of cancer care. PLS models could assist the cancer community in evaluating the (cost) effectiveness of cancer therapies, and due to their ability to take into account patient heterogeneity and history, these models may in some cases be more appropriate than conventional cohort models. Nevertheless, comprehensive comparisons with cohort models will be necessary to definitely ascertain the relative advantages and disadvantages of PLS models in the DPM domain. Additionally, PLS models may be used to better understand cancer care systems and to improve the delivery of care. The actual contribution of HCSO models to cancer care remains, however, unestablished and future studies will need to systematically assess and report the impact these models have on clinical practice. Finally, in both the DPM and HCSO domain more attention should be paid to the reporting quality of PLS models. In agreement with previous research (37), the assessment of papers in the present review revealed room for improvement in several areas, particularly with respect to model validation. Moreover, average reporting quality did not improve over time.
