# Supplementary material for: Patient-level simulation models in cancer care: a systematic review
Source: Front Public Health. 2025 May 9;13:1335300. doi: 10.3389/fpubh.2025.1335300 (PMC12098283; doi:10.3389/fpubh.2025.1335300)
Supplement: Supplementary file 1 [file Data_Sheet_1.docx]

**APPENDIX**

**1. Search strategy**

***1.1. PubMed***

(oncolog*[Title/Abstract] OR cancer[Title/Abstract] OR cancer*[Title/Abstract] OR neoplasm[Title/Abstract] OR neoplas*[Title/Abstract] OR Neoplasms[Mesh] OR malignancy[Title/Abstract] OR radiotherapy[Title/Abstract] OR Radiotherapy[Mesh] OR "radiation oncolog*"[Title/Abstract] OR chemoradiation [Title/Abstract] OR chemoradiotherapy [Title/Abstract] OR Chemoradiotherapy[Mesh] OR Neoadjuvant Therapy[Mesh] OR chemotherapy[Title/Abstract] OR Antineoplastic Protocols[Mesh] OR Induction Chemotherapy[Mesh] OR Maintenance Chemotherapy[Mesh] OR Consolidation Chemotherapy[Mesh] OR Chemotherapy, Adjuvant[Mesh] OR immunotherapy[Title/Abstract] OR Immunotherapy[Mesh] OR "targeted drugs"[Title/Abstract] OR "targeted therapy"[Title/Abstract] OR Molecular Targeted Therapy[Mesh] OR "hormone therapy"[Title/Abstract] OR ((surgery[Title/Abstract] OR General Surgery[Mesh] OR Surgical Procedures, Operative[Mesh]) AND (oncolog*[Title/Abstract] OR cancer[Title/Abstract] OR cancer*[Title/Abstract] OR neoplasm[Title/Abstract] OR Neoplasms[Mesh] OR neoplas*[Title/Abstract] OR malignancy[Title/Abstract]))) **AND** ("discrete event simulation"[Title/Abstract] OR "discrete events simulation"[Title/Abstract] OR ("discrete event"[Title/Abstract] AND ("computer simulat*"[Title/Abstract] OR "model*"[Title/Abstract])) OR microsimulation[Title/Abstract] OR "agent based model"[Title/Abstract] OR "agent based model*"[Title/Abstract] OR "individual based model"[Title/Abstract] OR "individual based model*"[Title/Abstract] OR "partially observable Markov decision process"[Title/Abstract] OR "individual sampling"[Title/Abstract] OR "patient level simulation"[Title/Abstract] OR "patient level model"[Title/Abstract] OR "patient level model*"[Title/Abstract])

***1.2. Web of Science***

(TS=(oncolog* OR cancer OR cancer* OR neoplasm OR neoplas* OR malignancy OR radiotherapy OR "radiation oncolog*" OR chemoradiation OR chemoradiotherapy OR chemotherapy OR immunotherapy OR "targeted drugs" OR "targeted therapy" OR "hormone therapy" OR (surgery AND (oncolog* OR cancer OR cancer* OR neoplasm OR neoplas* OR malignancy))) **AND** (TS=("discrete event simulation" OR "discrete events simulation" OR ("discrete event" AND ("computer simulat*" OR "model*")) OR microsimulation OR "agent-based model" OR "agent-based model*" OR "individual-based model" OR "individual-based model*" OR "partially observable Markov decision process" OR "individual sampling" OR "patient-level simulation" OR "patient-level model" OR "patient-level model*" ))

***1.3. Embase***

(oncolog*:ti,ab,kw OR ‘oncology'/exp OR cancer:ti,ab,kw OR cancer*:ti,ab,kw OR neoplasm:ti,ab,kw OR neoplas*:ti,ab,kw OR malignancy:ti,ab,kw OR 'malignant neoplasm'/exp OR radiotherapy:ti,ab,kw OR 'cancer radiotherapy'/exp OR chemotherapy:ti,ab,kw OR ‘radiation oncolog*’:ti,ab,kw OR chemoradiation:ti,ab,kw OR chemoradiotherapy:ti,ab,kw OR 'chemoradiotherapy'/exp OR 'cancer chemotherapy'/exp OR immunotherapy:ti,ab,kw OR 'cancer immunotherapy'/exp OR ‘targeted drugs’:ti,ab,kw OR ‘targeted therapy’:ti,ab,kw OR 'molecularly targeted therapy'/exp OR 'hormone therapy':ti,ab,kw OR 'cancer hormone therapy'/exp OR (surgery:ti,ab,kw AND ( oncolog*:ti,ab,kw OR cancer:ti,ab,kw OR cancer*:ti,ab,kw OR neoplasm:ti,ab,kw OR neoplas*:ti,ab,kw OR malignancy:ti,ab,kw)) OR 'cancer surgery'/exp) **AND** ('discrete event simulation':ti,ab,kw OR 'discrete events simulation':ti,ab,kw OR ('discrete event':ti,ab,kw AND ('computer simulat*':ti,ab,kw OR 'model*':ti,ab,kw)) OR 'discrete event simulation'/exp OR microsimulation:ti,ab,kw OR 'microsimulation'/exp OR 'agent-based model':ti,ab,kw OR 'agent-based model*':ti,ab,kw OR 'agent based model'/exp OR 'agent based modeling'/exp OR 'individual-based model':ti,ab,kw OR 'individual-based model*':ti,ab,kw OR 'individual based model'/exp OR 'individual based population model'/exp OR 'partially observable Markov decision process':ti,ab,kw OR 'individual sampling':ti,ab,kw OR 'patient-level simulation':ti,ab,kw OR 'patient-level model':ti,ab,kw OR 'patient-level model*':ti,ab,kw)

***1.4. Econlit***

(oncolog* OR cancer OR cancer* OR neoplasm OR neoplas* OR malignancy OR radiotherapy OR chemotherapy OR immunotherapy OR hormone therapy OR (surgery AND (oncolog* OR cancer OR cancer* OR neoplasm OR neoplas* OR malignancy))) **AND** ("discrete event simulation" OR "discrete events simulation" OR ("discrete event" AND ("computer simulat*" OR "model*")) OR microsimulation OR "agent-based model" OR "agent-based model*" OR "individual-based model" OR "individual-based model*" OR "partially observable Markov decision process" OR "individual sampling" OR "patient-level simulation" OR "patient-level model" OR "patient-level model*" ))))

**Description of modelling techniques**

***State-transition microsimulation (STMS)***

As explained by the ISPOR-SMDM taskforce [1], state-transition microsimulation (STMS) models resemble cohort state-transition models in the sense that they are both structured around a set of mutually exclusive and collectively exhaustive health states. In a STMS model, however, patients progress through the model one-by-one in a probabilistic manner rather than as a single homogeneous cohort. STMS models overcome some important limitations of cohort models because they can take into account each patient’s individual characteristics and history [2]. STMS modelling has a long history as a tool for (health) policy evaluation [3] and has, for example, regularly been employed to examine the effects of cancer screening programmes [4].

***Discrete event simulation (DES)***

Discrete event simulation (DES) finds its origin in operations research, although the technique is now increasingly used within healthcare management and health economics [5, 6]. As the ISPOR-SMDM Taskforce demonstrates[7], DES can be best explained in terms of its key concepts, which are entities, attributes, events, resources, queues, and time (see also [8]. Entities are objects which can interact with other entities and the wider system. Within healthcare models entities are typically patients but there is in principle no limit to what entities can represent (e.g., other people, things, places). Entities experience events (e.g., cancer recurrence, adverse effects, treatment switching) and have attributes (e.g., age, quality of life, past events) which determine how an entity responds and which are flexibly updated over the course of the simulation. Consequently, individual differences and memory can be efficiently incorporated into the model. Resources (e.g., doctors, linear accelerators) provide services to entities, which can require time. If a resource is occupied when an entity calls on it, the entity will need to wait and a queue will form. Queues have logic (e.g., first-in-first-out, selection based on priority) and can have a maximum capacity. By explicitly modelling resources, DES permits users to analyse the effects of resource constraints. Furthermore, contrary to Markov models, time does not progress in fixed time steps in a DES model but efficiently advances to the next event time. Events are thus allowed to occur at any time point without any superfluous calculations in-between.

***Agent-based models***

Agent-based models represent an extension of DES [7, 9]. Agents interact with others and their environment according to a set of rules that describe their behaviour. With respect to cancer, agent-based modelling has been used to construct biological models of tumours and interacting cells [10-12].

***Timed automata***

Timed automata is a technique originating from computer science to model distributed systems over time. Degeling et al. [13] demonstrate that timed automata is well-suited to model personalised treatments, yet the need for specific software (i.e., UPPAAL) limits its usefulness and applications in health care are at the moment very rare.

**Table A1: DPM models**

| Paper | Year | Model | Category | Cancer | WBC | Software | Sensitivity analysis | Therapeutic options | Results |
| --- | --- | --- | --- | --- | --- | --- | --- | --- | --- |
| [14] | 2010 | STMS | CE | DLBCL | HIC | UnS | PSA | (1) CHOP; (2) CHOP-R | CHOP-R is a cost-effective alternative to CHOP for 1L treatment, particularly in individuals older than 60. |
| [15] | 2011 | STMS | CE | Prostate (metastatic) | HIC | TreeAge Pro | PSA | (1) Leuprorelin 11.25 mg; (2) Leuprorelin 22.5 mg; (3) Triptorelin 11.25 mg; (4) Buserelin 9.9 mg; (5) Goserelin 10.8 mg | Leuprorelin 22.5 mg is the most cost-effective treatment. |
| [16] | 2011 | DES | CE | Multiple myeloma  (relapsed refractory) | HIC | Rockwell Arena | DSA | (1) Lenalidomide + dexamethasone; (2) Bortezomib | Lenalidomide + dexamethasone is the most cost-effective. |
| [17] | 2012 | DES | CE | Prostate  (localised) | HIC | Simul8 | DSA & PSA | (1) IMRT; (2) 3DCRT | CE of IMRT is uncertain, if it can prolong survival it is cost-effective. |
| [18] | 2012 | STMS | CE | NHL  (relapsed) | HIC | TreeAge Pro | DSA & PSA | (1) Granulocyte colony-stimulating factor + plerixafor; (2) Granulocyte colony-stimulating factor | Granulocyte colony-stimulating factor + plerixafor for stem cell mobilization is cost-effective. |
| [19] | 2013 | STMS | CE | Lung, colorectal | HIC | UnS | None | Generic model | Model can project population health and economic impacts of cancer control programs in Canada and impacts of major risk factors, cancer prevention, screening programs and new cancer treatments on population health and healthcare system costs. |
| [20] | 2013 | DES | CE | Multiple myeloma (relapsed refractory) | HIC | Rockwell Arena | DSA & PSA | (1) Lenalidomide + dexamethasone; (2) Bortezomib | Lenalidomide + dexamethasone is most cost-effective. |
| [21] | 2013 | STMS | HO  (QALYs) | Colorectal  (T1 adenocarcinoma low rectum) | UnS | R | DSA | (1) Transanal local excision; (2) Abdominoperineal resection | Transanal local excision was the preferable approach for most patients. Minority group in whom abdominoperineal resection is preferred are those who are unwilling to sacrifice 7% of their LE to avoid a permanent stoma. |
| [22] | 2013 | DES | CE | Leukemia (acute myeloid) | HIC | Microsoft Excel (with macros programmed in Visual Basic for Applications), SAS | PSA | Generic model | The model represents the full disease course from diagnosis and can be used in CE analyses of new genetic tests and chemotherapy treatments. |
| [23] | 2013 | DES | HO  (disease-free, invasive disease-free, and overall survival and breast preservation) | Breast  (DCIS) | HIC | TreeAge Pro | DSA | (1) Lumpectomy; (2) Lumpectomy + radiotherapy; (3) Lumpectomy + radiotherapy + tamoxifen; (4) Lumpectomy + tamoxifen; (5) Mastectomy with reconstruction; (6) Mastectomy without reconstruction | Overall survival benefits of the 6 management strategies are within 1 year, suggesting that treatment decisions can be informed by the patient’s preference for breast preservation and disutility for recurrence. |
| [24] | 2013 | DES | CE | Colorectal | HIC | Simul8 | PSA | Generic model | Whole Disease Model development is feasible and can allow for the economic analysis of most interventions across a disease service within a consistent conceptual and mathematical infrastructure. The model was capable of evaluating 11 of 15 guideline topics, ranging from alternative diagnostic technologies through to treatments for metastatic disease. |
| [25] | 2013 | STMS | HO  (LE gains) | Lung  (Inoperable Stage I NSCLC) | HIC | UnS | DSA | (1) Radio-frequency ablation; (2) SBRT; (3) Radiotherapy | Radio-frequency ablation and SBRT could provide LE gains compared with radiotherapy. |
| [26] | 2014 | DES | CE | NHL  (advanced indolent) | HIC | R | DSA & PSA | (1) Bendamustine-rituximab; (2) CHOP-R; (3) CVP-R | Bendamustine-rituximab is cost-effective. |
| [27] | 2014 | STMS | Costs  (drug costs) | Prostate (metastatic castration-resistant) | HIC | TreeAge Pro | DSA | (1) Primary sequence (bicalutamide; bicalutamide withdrawal; docetaxel based-chemotherapy + prednisone; abiraterone acetate + prednisone; OtherTx.); (2) Alternate sequence (bicalutamide; bicalutamide withdrawal; abiraterone acetate + prednisone; docetaxel based-chemotherapy + prednisone; cabazitaxel based-chemotherapy plus prednisone; OtherTx.) | The cost of medications is significant and susceptible to increase in the near future to prohibitive levels. |
| [28] | 2014 | STMS | HO  (LE, QALE) | Hodgkin lymphoma (advanced stage) | UnS | UnS | DSA | (1) Secondary prophylaxis with granulocyte colony-stimulating factor; (2) No granulocyte colony-stimulating factor | There was a net benefit of 0.017 years and 0.037 quality-adjusted LYs for not using granulocyte colony-stimulating factor. |
| [29] | 2014 | DES | CE | Ovarian (advanced stepithelial) | HIC | Rockwell Arena | DSA & PSA | (1) Early initiated treatment (Preliminary diagnosis and tests; exploratory laparoscopy; surgery or first cycle of chemotherapy; primary treatment); (2) Current treatment (cfr. 1) | Early-initiated treatment is cost-effective. |
| [30] | 2014 | DES | CE | Leukemia (chronic lymphocytic) | HIC | UnS | DSA & PSA | (1) Bendamustine; (2) Alemtuzumab; (3) Chlorambucil | Bendamustine is cost-effective. |
| [31] | 2014 | STMS | CE | Lung  (stage I NSCLC) | HIC | UnS | None | Introduction SBRT in Canadian Cancer Risk Management model. | SBRT dominated radiotherapy, sublobar resection, and best supportive care. |
| [32] | 2015 | DES | CE | Colorectal (stage-III colon) | HIC | TreeAge Pro | DSA | (1) Cancer care coordinator; (2) business as usual. | Cancer care coordinators are cost-effective. |
| [33] | 2015 | DES | CE | Breast  (ER and/or PR positive, HER-2/neu negative, lymph node negative) | HIC | Rockwell Arena | DSA | 8 test-treatment strategies (tests: OncotypeDX and Adjuvant! Online, treatment: chemotherapy) | OncotypeDX applied in all risk groups is the most effective strategy. |
| [34] | 2015 | STMS | CE | CNS  (brain metastases) | HIC | TreeAge Pro | DSA & PSA | (1) Whole-brain radiotherapy; (2) Stereotactic radiosurgery + whole-brain radiotherapy salvage; (3) Hippocampal avoidance whole-brain radiotherapy; (4) Stereotactic radiosurgery + stereotactic radiosurgery salvage; (5) Stereotactic radiosurgery + hippocampal avoidance whole-brain radiotherapy salvage; (6) Stereotactic radiosurgery + whole-brain radiotherapy; (7) Stereotactic radiosurgery + hippocampal avoidance whole-brain radiotherapy | Traditional radiation therapies are cost-effective for patients in cohorts with a median survival of 3 - 6 months. In cohorts with longer median survival, hippocampal avoidance whole-brain radiotherapy and stereotactic radiosurgery + hippocampal avoidance whole-brain radiotherapy are cost-effective relative to traditional treatments. When the treatments that involved hippocampal avoidance whole-brain radiotherapy are excluded, either stereotactic radiosurgery alone or stereotactic radiosurgery + whole-brain radiotherapy is cost-effective relative to whole-brain radiotherapy alone. |
| [35] | 2016 | DES | CE | Lung  (advanced NSCLC) | LMIC | TreeAge Pro | DSA & PSA | (1) EGFR-mutation testing + appropriate treatment (Gefitinib in case of mutation); (2) Chemotherapy | EGFR-mutation testing + appropriate treatment is cost-effective. |
| [36] | 2016 | DES | CE | Multiple myeloma (elderly) | HIC | UnS | DSA & PSA | 2 real world treatment patterns with 3 lines of therapy and 4 alternative scenarios (agents used: melphalan–prednisone, thalidomide, bortezomib, lenalidomide & other) | The sequence thalidomide - lenalidomide - bortezomib is most cost-effective. |
| [37] | 2016 | DES | CE | Multiple myeloma | HIC | UnS | DSA | (1) Pomalidomide + low-dose dexamethasone; (2) High-dose dexamethasone | Pomalidomide is associated with a relatively high incremental cost per QALY gained (84.900 euro). |
| [38] | 2016 | STMS | CE | Bone  (painful bone metastases in breast, lung and prostate cancer) | HIC | TreeAge Pro | DSA | (1) Single; (2) Multiple fraction palliative radiotherapy | Single is more cost-effective than multiple fraction palliative radiotherapy for all 3 cancers. |
| [39] | 2016 | STMS | HO  (QALYs) | Breast  (women with first-, second-degree, or no family history and stage I, II, or III ER-positive or ER-negative breast cancer) | HIC | TreeAge Pro | DSA & PSA | (1) Contralateral prophylactic mastectomy; (2) Surveillance | Women age 40 with stage I breast cancer and a first-degree relative with bilateral breast cancer have a QALY benefit from contralateral prophylactic mastectomy similar to that for BRCA1/2mutation carriers. For most subgroups, contralateral prophylactic mastectomy has a minimal to no effect on QALE, irrespective of family history. |
| [40] | 2016 | STMS | CE | Leukemia (childhood precursor B-cell acute lymphoblastic) | HIC | TreeAge Pro | DSA & PSA | (1) Minimal residual disease testing by flow cytometry; (2) No testing | Minimal residual disease testing by flow cytometry is cost-effective. |
| [41] | 2016 | DES | CE | Breast | HIC | Rockwell Arena | DSA & PSA | 8 test-treatment strategies (tests: OncotypeDX and Adjuvant! Online, treatment: chemotherapy) | Testing all patients is cost-effective. |
| [42] | 2016 | DES | HO  (QALYs) | Head & neck | HIC | AnyLogic | DSA | (1) Proton; (2) Photon radiotherapy | The model can distinguish between patients with high and low potential benefits from proton therapy. Benefits are highest for patients with both good prognosis and high expected damage to adjacent organs. |
| [43] | 2016 | STMS | HO  (30-day all-cause mortality and mean survival duration) | Poor-prognosis primary tumors, metastatic disease, hematologic malignant neoplasms | HIC | TreeAge Pro, R | DSA & PSA | Thirty-one treatment strategies (time-unlimited aggressive care, CMO, and 29 time-varying intensive care unit trial strategies) | Trials of ICU care lasting 1 to 4 days may be sufficient in patients with poor-prognosis solid tumors, whereas patients with hematologic malignant neoplasms or less severe illness seem to benefit from longer trials of intensive care. |
| [44] | 2016 | STMS | CE | Lung  (early NSCLC) | HIC | TreeAge Pro | DSA & PSA | (1) myPlan Lung Cancer prognostic test; (2) Standard of care | Testing is potentially cost-effective based on globally accepted WTP thresholds. |
| [45] | 2017 | STMS | HO  (prostate cancer mortality, overtreatment) | Prostate | HIC | UnS | DSA & PSA | (1) Basecase yearly screening; (2) Yearly screening + active surveillance (AS) for ≤ T1GS6 men, with yearly biopsies; (3) Yearly screening + AS for ≤ T2aGS6 men, with yearly biopsies; (4) Yearly screening + AS for ≤ T2aGS6 men, with biannual biopsies after 1st year; (5) Yearly screening + AS for ≤ T2aGS6 men, with triannual biopsies after 1st year; (6) Yearly screening + AS for ≤ T2aGS6 men, with biopsy every 5 years after 1st year; (7) Yearly screening, AS for ≤ T2aGS7 men, with yearly biopsies. | Active surveillance for low-risk patients is relatively safe. Increasing the biopsy interval from yearly to up to every 3 years after the first year, will significantly reduce overtreatment among low-risk men, with limited prostate cancer mortality risk. |
| [46] | 2017 | STMS | CE | Prostate | HIC | UnS | DSA | AS under different biopsy follow-up schedules (1, 4, 7, or 10 yearly biopsy rounds) vs conservative management | The benefit of AS compared to conservative management is strongly dependent on LE and disease risk. For men with low-risk disease in younger age groups (55-65), AS is cost-effective for up to 7 yearly biopsy rounds. For men older than 65, even one biopsy round results QALYs lost, though it may result in QALYs gained for men without previous screening. For men with intermediate-risk disease AS is cost-effective even for men in 65-75 age group. |
| [36] | 2017 | DES | CE | Kidney (metastatic renal cell carcinoma) | HIC | UnS | DSA & PSA | (1) Real-world treatment mix; (2) No targeted therapy; (3) Real-world mix + sunitinib for eligible patients (followed by sorafenib); (4) Real-world mix + sunitinib for eligible patients (followed by everolimus); (5) Real-world mix + sunitinib for eligible patients (followed by other) | Health can be gained if more treatment-eligible patients receive targeted therapies and it will be just as cost-effective to treat these patients with sunitinib as current treatment practice. |
| [13] | 2017 | DES, TA | CE | Prostate (metastatic castration-resistant) | HIC | AnyLogic (DES), UPPAAL (TA) | DSA | Use of (1) Circulating tumor cells; (2) PSA; (3) Bone scintigraphy as response marker | Applying circulating tumor cell as a response marker reduces overtreatment by 6.99 and 7.02 weeks at a net monetary benefit of €1033 and €1104 for TA and DES. |
| [47] | 2017 | STMS | CE | Leukemia (acute myeloid with normal karyotype) | HIC | TreeAge Pro | DSA | (1) Conventional cytogenetic diagnostics; (2) Molecular genetic diagnostics | Molecular genetic diagnostics is cost-effective with an (ICER) of about US$ 4928 per survived month. |
| [48] | 2017 | DES | CE | Breast | HIC | Rockwell Arena | DSA | 8 test-treatment strategies (tests: OncotypeDX and Adjuvant! Online, treatment: chemotherapy) | Chemotherapy is effective and cost effective for Austrian patients with an intermediate/high Adjuvant! Online risk and an intermediate/high OncotypeDX risk. |
| [49] | 2017 | DES | CE | Breast (hormone receptor-positive advanced) | HIC | Rockwell Arena | DSA & PSA | Endocrine treatment naive patients ((1) Palbociclib + letrozole; (2) Letrozole) and patients with prior endocrine therapy ((1) Palbociclib + fulvestrant; (2) Fulvestrant) | Palbociclib for both patients with as without prior endocrine therapy is highly unlikely to be cost-effective compared with the usual care in the USA. |
| [50] | 2017 | DES | CE | Colorectal (elderly metastatic) | HIC | TreeAge Pro | DSA & PSA | (1) 1L oxaliplatin/ irinotecan; 2L oxaliplatin/ irinotecan + bevacizumab; (2) 1L oxaliplatin/ irinotecan + bevacizumab; 2L oxaliplatin/ irinotecan + bevacizumab; (3) 1L oxaliplatin/ irinotecan; 2L oxaliplatin/ irinotecan + bevacizumab; 3L targeted biologic; (4) 1L oxaliplatin /irinotecan + bevacizumab; 2L oxaliplatin /irinotecan + bevacizumab; 3L targeted biologic | Survival increases marginally with the addition of targeted biologics, such as bevacizumab, at first line and third line at substantial costs. Treatment sequences with bevacizumab at first line and targeted biologics at third line may not be cost-effective at the commonly used threshold of $100,000/QALY gained, but a marginal decrease in the cost of bevacizumab may make sequences with 1L bevacizumab cost-effective. |
| [51] | 2017 | DES | CE | Breast (hormone receptor-positive advanced) | UnS | TreeAge Pro | PSA | (1) Palbociclib + letrozole; (2) Letrozole | Palbociclib for patients with & without prior endocrine therapy is highly unlikely to be cost-effective compared with usual care in the USA. |
| [52] | 2017 | STMS | CE | Head and neck (oropharyngeal squamous cell carcinoma) | HIC | TreeAge Pro | DSA & PSA | (1) Radiotherapy; (2) Trans-oral robotic surgery | Trans-oral robotic surgery offered positive health benefits compared to radiotherapy, but at a substantially higher cost, and would not be cost-effective in the U.S. Trans-oral robotic surgery may be a cost-effective alternative in early-stage patients, but demonstrates considerable sensitivity to assumptions around quality of life. |
| [53] | 2017 | DES | HO & CO  (LE; treatment cost) | DLBCL | HIC | Simul8 | None | Generic model | The model simulates and predict treatment costs and LY gained throughout the treatment pathway. The model produces different outputs for different purposes; estimating total costs along with health benefits at varying time points for specific patient cohorts, as well as generating prevalence-based costs for all patients over specific time periods. |
| [54] | 2018 | STMS | HO  (mortality reduction) | Breast | LMIC | R | None | Three early detection strategies alone or in combination with three systemic treatment programmes beyond standard of care (programme A): programme B was endocrine therapy for all oestrogen-receptor (ER)-positive cases; programme C was programme B plus chemotherapy for ER-negative cases; programme D was programme C plus chemotherapy for advanced ER-positive cases | The best projected outcomes were in settings where access to both early detection and adjuvant therapy is improved. Even in the absence of mammographic screening, improvements in detection can provide substantial benefit in settings where advanced-stage presentation is common. |
| [55] | 2018 | STMS | CE | DLBCL | HIC | UnS | PSA | (1) R-CHOP for all patients; (2) Subtype testing followed by R-CHOP for germinal center B-cell-like DLBCL and novel treatment for Activated B-cell-like DLBCL; (3) Novel treatment for all patients | The subtype-based approach showed a favorable incremental CE ratio of $15,015/QALY compared with R-CHOP. |
| [56] | 2018 | DES | CE | Colorectal (metastatic) | HIC | Anylogic | PSA | (1) CAP-B maintenance treatment (capecitabine + bevacizumab); (2) Observation until progression | CAP-B maintenance treatment is not cost-effective. |
| [57] | 2018 | DES | HO  (overall survival) | Prostate (metastatic castration-resistant) | HIC | Microsoft Excel | None | (1) Abiraterone acetate + prednisone; (2) Prednisone | Abiraterone acetate + prednisone yields greater overall survival. |
| [58] | 2018 | DES | CE | Skin  (BRAF wild-type advanced melanoma) | HIC | Microsoft Excel, Visual Basic for Applications | DSA & PSA | 4 treatment sequences with checkpoint inhibitors ((1) 1L Anti-CTLA-4; 2L Anti-PD-1; 3L Chemotherapy or best supportive care; (2) 1L Anti-PD-1; 2L Anti-CTLA-4; 3L Chemotherapy or best supportive care; (3) 1L Anti-PD-1 + anti-CTLA-4; 2L Chemotherapy; 3L Chemotherapy or best supportive care; (4) 1L Anti-PD-1 + anti-CTLA-4; 2L Anti-PD-1; 3L Chemotherapy or best supportive care | Anti-PD-1 + anti-CTLA-4 initiating sequences are cost-effective versus anti-PD-1. |
| [59] | 2018 | DES | HO & CO  (LE, QALYs; lifetime & annual treatment costs) | Follicular lymphoma | HIC | Simul8 | PSA | Generic model | Costs, survival, and QALYs vary markedly with patient characteristics and disease management. Allowing the production of more realistic outcomes across the patient population as a whole, the model addresses this heterogeneity and is a useful tool with which to evaluate new technologies/treatments to support healthcare decisionmakers. |
| [60] | 2018 | STMS | HO  (LE gains) | Ovarian (advanced) | HIC | TreeAge Pro | DSA | (1) Triage between primary cytoreductive surgery and neoadjuvant chemotherapy followed by interval cytoreductive surgery based on current clinical practice; (2) based on hypothetical test. | Dagnostic tests designed to triage patients will likely have only a modest effect on life expectancy. |
| [61] | 2019 | STMS | CE | CNS  (high-grade gliomas) | HIC | TreeAge Pro | DSA & PSA | (1) High-grade glioma resection with intraoperative MRI; (2) High-grade glioma resection without intraoperative MRI | Intraoperative MRI is likely to be cost-effective. |
| [62] | 2019 | STMS | CE | Lung  (stage III NSCLC) | HIC | UnS | DSA | (1) Durvalumab; (2) No consolidation therapy | Durvalumab consolidation therapy is cost-effective despite having a substantial budgetary consequence. |
| [63] | 2019 | STMS | CE | Lung  (metastatic nonsquamous NSCLC) | HIC | TreeAge Pro | DSA & PSA | (1) Bevacizumab + carboplatin + paclitaxel; (2) Atezolizumab + bevacizumab + carboplatin + paclitaxel; (3) Carboplatin + pemetrexed; (4) Pembrolizumab + carboplatin + pemetrexed | Atezolizumab combination was not cost-effective compared with bevacizumab, carboplatin, and paclitaxel and provided suboptimal incremental benefit compared with cost vs pembrolizumab combination for 1L treatment. |
| [64] | 2019 | STMS | CE | Bladder (advanced urothelial carcinoma of the bladder) | HIC | UnS | DSA | (1) Standard-of-care chemotherapy for all patients; (2) Pembrolizumab for all patients; (3) Pembrolizumab for patients with PD-L1-positive tumors at a ≥1% expression threshold and chemotherapy for all others | Pembrolizumab was not cost-effective in either strategy based on a $100,000/QALY WTP threshold. Using PD-L1 testing to select for patients who may have better associated outcomes may improve the affordability of pembrolizumab. |
| [65] | 2019 | DES | CE | Breast  (early HER2 positive) | HIC | Microsoft Excel + Ersatz add-in | DSA & PSA | Trastuzumab | The ICER was A$132,537 per DALY averted. Restricting trastuzumab to women aged 40 years or younger with tumour sizes 40+ mm reduced the ICER to A$35,290 per DALY averted. |
| [66] | 2019 | STMS | CE | CNS  (pediatric medulloblastoma) | LMIC | TreeAge Pro | DSA & PSA | (1) Proton; (2) Photon radiotherapy | Proton therapy is not cost-effective from the Brazilian health system perspective. |
| [67] | 2019 | STMS | CE | Skin (metastatic melanoma) | HIC | Microsoft Excel | None | (1) Nivolumab + ipilimumab; (2) Ipilimumab; (3) Nivolumab | Combinationtherapy yields an ICER of £6,474 per QALY gained. |
| [68] | 2019 | STMS | CE | Breast  (early-stage node-negative, HER2-positive) | HIC | MATLAB | DSA & PSA | (1) Adjuvant paclitaxel + trastuzumab; (2) Doxorubicin + cyclophosphamide + paclitaxel + trastuzumab; (3) Docetaxel + carboplatin + trastuzumab; (4) No adjuvant trastuzumab | Adjuvant paclitaxel + trastuzumab is cost-effective for all age groups. |
| [69] | 2019 | STMS | CE | Leukemia (chronic myeloid) | HIC | UnS | DSA & PSA | (1) Imatinib mesylate with therapeutic drug monitoring; (2) Imatinib mesylate without therapeutic drug monitoring | Therapeutic drug monitoring is cost-effective. |
| [70] | 2019 | STMS | HO  (LE gains) | Ovarian (advanced) | HIC | TreeAge Pro | DSA | (1) Optimized; (2) current implementation of standard intravenous chemotherapy; intraperitoneal + intravenous chemotherapy; bevacizumab + intravenous chemotherapy; and hyperthermic intraperitoneal chemotherapy + intravenous chemotherapy. | LE can be substantially improved by optimized implementation (LE gain = 12.2 months). |
| [71] | 2019 | STMS | CE | Leukemia (pediatric relapsed/refractory B-cell acute lymphoblastic) | HIC | TreeAge Pro | DSA & PSA | (1) CAR-T therapy; (2) Standard care | CAR-T therapy is cost-effective but follow-up to assess long-term outcomes is required to confirm the validity of these preliminary findings. |
| [72] | 2019 | STMS | CE | Breast, lung, gynecological, non-Hodgkin lymphoma. | HIC | UnS | DSA & PSA | (1) Current use of granulocyte colony-stimulating factor; (2) Targeted use of granulocyte colony-stimulating factor; (3) Reduced use of granulocyte colony-stimulating factor | Current use of G-CSF prophylaxis would provide $96 billion in social value over the next 10 years. Targeting G-CSF prophylaxis to align with guidelines would more than double social value. |
| [73] | 2019 | DES | CE | Skin (BRAF-mutant advanced melanoma) | HIC | UnS | DSA | (1) 1L anti-PD-1 + anti-CTLA-4; 2L BRAF + MEK inhibitors; (2) 1L anti-PD-1; 2L BRAF + MEK inhibitors; (3) 1L BRAF + MEK inhibitors; 2L anti-PD-1 | Initiating treatment with anti-PD-1+anti-CTLA-4 is more cost–effective than initiation with anti-PD-1 monotherapy or BRAF+MEK inhibitors. |
| [74] | 2019 | DES | HO  (1-year all-cause mortality) | DLBCL | HIC | R | None | Nine scenarios of wait times ranging from 1 to 9 months for chemotherapy or for CAR T-cells. | Increasing wait time of receiving CAR T-cell therapy from 1 to 9 months increased predicted 1-year mortality rate from 36.1% to 76.3%. Baseline 1-year mortality was 34.0% in patients receiving CAR T-cell therapy with no wait times and 75.1% in patients treated with chemotherapy. This resulted in an increased relative mortality rate of 6.2% to 124.5% over a 1- to 9-month wait time compared with no wait time. |
| [75] | 2019 | DES | CE | Breast (hormone-receptor-positive advanced) | LMIC | TreeAge Pro | PSA | (1) Ribociclib + letrozole; (2) Placebo + letrozole | Ribociclib is cost-effective at a price of $732 or $1170 per 4 weeks for China and Beijing City, respectively. |
| [76] | 2019 | STMS | CE | Breast  (low-risk early, age 70 or above) | UnS | TreeAge Pro, R | DSA & PSA | (1) 5 years of anastrozole; (2) 15-fraction course of radiation without boost | Patients who are reluctant or unable to pursue adjuvant AI can safely pursue adjuvant radiation with limited differences in outcome and modest increase in costs. |
| [77] | 2019 | STMS | HO  (5-year net survival) | Pediatric | UnS | Java | None | Increasing availability of treatments (chemotherapy, radiation, general surgery, neurosurgery, ophthalmic surgery), reducing treatment abandonment, and improving the quality of care to mean of HIC. | Although expanding access to treatment (chemotherapy, radiation, and surgery) and addressing financial toxicity are essential, investments that improve the quality of care, at both the health-system and facility level, are needed to improve outcomes globally. |
| [78] | 2019 | STMS | CE | Prostate  (low risk) | HIC | TreeAge Pro, R | DSA & PSA | (1) Immediate curative treatment; (2) Low-intensity AS; (3) Modified low-intensity AS; (4) Medium-intensity AS; (5) High-intensity AS | For a 65-year-old man AS with biennial biopsy is highly cost-effective compared with common alternatives. AS with triennial biopsy dominates all other strategies and should be considered for men who are comfortable with a longer period between biopsies. Optimal strategy depends on tolerance for periodic biopsies and comfort with delaying radical treatment. |
| [79] | 2020 | DES | CE | Kidney (intermediate-to poor-risk advanced renal cell carcinoma) | HIC | UnS | DSA & PSA | (1) 1L nivolumab + ipilimumab; 2L cabozantinib, axitinib, pazopanib or lenvatinib + everolimus (2) 1L cabozantinib; 2L nivolumab, axitinib, pazopanib or lenvatinib + everolimus; (3) 1L sunitinib or pazopanib: 2L nivolumab, cabozantinib, axitinib or lenvatinib + everolimus. | Sequences initiating with nivolumab + ipilimumab resulted in the highest survival gain compared with sequences initiating with TKI. ICER per QALY gained for nivolumab + ipilimumab initiating sequences were $16,524–$125,860 compared with the TKI-initiating sequences. |
| [80] | 2020 | DES | HO  (QALYs) | Prostate  (AS-eligible with kidney failure) | UnS | R (simmer package) | PSA | (1) Definitive treatment & listing after waiting period of 2 years; (2) Definitive treatment & immediate listing; (3) AS & listing after waiting period of 2 years; (4) AS & immediate listing | AS and immediate listing outperformed alternative strategies from a QALE perspective, followed by definitive treatment and immediate listing. |
| [81] | 2020 | STMS | CE | Breast  (with lymphedema) | HIC | TreeAge Pro | DSA | (1) Conservative management; (2) Autologous abdominally-based free flap breast reconstruction; (3) Breast reconstruction + VLNT | Combining delayed breast reconstruction with VLNT in patients with existing lymphedema is cost-effective. |
| [82] | 2020 | STMS | CE | Lung  (advanced NSCLC) | HIC | UnS | DSA | Base case patients ((1) Pembrolizumab + chemotherapy; (2) Chemotherapy); only patients with PD-L1 expression ≥50% ((1) Pembrolizumab + chemotherapy; (2) Chemotherapy; (3) Pembrolizumab) | 1L treatment with pembrolizumab is not cost-effective compared to chemotherapy. |
| [83] | 2020 | DES | HO (progression-free & overall survival) | Colorectal (metastatic) | HIC | R | PSA | (1) Doublet chemotherapy; (2) Doublet chemotherapy + bevacizumab | A simulation utilizing real world data can be used to identify the optimal combination of first line treatment options for specific patient subpopulations. |
| [84] | 2020 | STMS | CE | Prostate (metastatic hormone-sensitive) | HIC | TreeAge Pro | DSA | (1) Androgen deprivation therapy + abiraterone acetate; (2) Androgen deprivation therapy + docetaxel | Although androgen deprivation therapy + abiraterone acetate results in a gain in QALYs and crude overall survival compared to docetaxel, abiraterone acetate therapy is not a cost-effective treatment strategy to apply uniformly to all patients. |
| [85] | 2020 | DES | CE | Breast | HIC | Rockwell Arena | PSA | (1) Oncotype genomic test to inform decision to use chemotherapy or not; (2) Standard of treatment based on clinical-pathological criteria | Oncotype is cost-effective from a health system perspective since each QALY gained costs less than 25,000 euros. From a societal perspective, it is dominant since it provides greater health and is accompanied by cost savings. |
| [86] | 2020 | STMS | HO  (metastasis risk, mortality risk, LYs, QALYs) | Prostate | HIC | UnS | DSA | 9 fixed biopsy schedules and 3 risk-tailored biopsy schedules | Among men diagnosed with GS ≤6 prostate cancer, obtaining a biopsy every 3 or 4 years appears to be an acceptable alternative to more frequent biopsies. Reducing AS intensity for those who have a low risk of progression reduces the number of biopsies while preserving the benefit of more frequent schedules. |
| [87] | 2020 | STMS | Costs  (budget impact) | Lung  (NSCLC) | HIC | UnS | DSA | (1) 1L pembrolizumab; (2) 2L pembrolizumab; (3) Nivolumab for nonsquamous NSCLC; (4) Nivolumab for squamous NSCLC | Anti-PD-1 agents are associated with a substantial economic burden (€373.1 million in 2019 in France). |
| [88] | 2020 | DES | HO & CO (median survival, 5-year survival rate; life-time per-treated-patient cost) | Lung  (advanced NSCLC) | HIC | Microsoft Excel | None | Generic model | The iTEN model is a reliable tool for forecasting the impact on cost and survival of new treatments. |
| [89] | 2020 | STMS | CE | Ovarian  (primary epithelial, BRCA variant or HRD without BRCA variant or HRD | HIC | TreeAge Pro | None | BRCA variant ((1) Olaparib; (2) Olaparib + bevacizumab (3) Bevacizumab; (4) Niraparib; (5) No maintenance therapy); HRD & HRD without BRCA variant ((1) Olaparib + bevacizumab; (2) Bevacizumab; (3) Niraparib; (4) No maintenance therapy) | At current costs, maintenance therapy for primary ovarian cancer is not cost-effective, regardless of molecular signature. |
| [90] | 2020 | STMS | CE | Lung  (advanced NSCLC) | LMIC | UnS | None | (1) RT-PC test for EGFR; FISH test for ALK; FISH test for ROS1; (2) RT-PC test for EGFR; FISH test for ALK and ROS1 simultaneously; (3) Single test next-generation sequencing, platform that includes EGFR, ALK and ROS1 genes | Molecular diagnosis by next-generation sequencing was not cost-effective in terms of QALYs from the perspective of the Brazilian supplementary health system. |
| [91] | 2020 | STMS | CE | Breast  (low-risk hormone-positive early-stage, women aged ≥ 70) | HIC | TreeAge Pro, R | DSA & PSA | (1) AI for 5 years; (2) 5-fraction course of APBI using IMRT; (3) Combination of both | Both AI-alone and APBI-alone are reasonable options but the combination is only cost-effective in a minority of scenarios. |
| [92] | 2020 | STMS | HO  (5-year net survival) | Cervical | UnS | Java | DSA & PSA | Scaling up imaging (ultrasound, x-ray, CT, MRI, PET, SPECT), treatment (chemotherapy, radiotherapy, surgery, targeted therapy) & quality of care to mean level of HIC, individually & in combination. | Scale-up of treatment, imaging, and quality of care could substantially improve global cervical cancer 5-year net survival, with quality of care and imaging improvements each contributing about 25% of the total potential gains. |
| [93] | 2020 | STMS | HO  (5-year net survival) | Multiple  (oesophagus, stomach, colon, rectum, anus, liver, pancreas, lung, breast, cervix uteri, prostate) | UnS | Java | DSA & PSA | Scaling up imaging (ultrasound, x-ray, CT, MRI, PET, SPECT), treatment (chemotherapy, radiotherapy, surgery, targeted therapy) & quality of care to mean level of HIC, individually & in combination. | Scaling up treatment and imaging availability could yield synergistic survival gains. Expanding traditional modalities in lower-income settings might be a feasible pathway to improve survival before scaling up more modern technologies |
| [94] | 2020 | STMS | CE | Kidney  (advanced renal cell carcinoma) | HIC | TreeAge Pro | DSA & PSA | Intermediate- & poor-risk population ((1) 1L pembrolizumab + axtinib; (2) 1L nivolumab + ipilimumab) and Favorable-Risk population ((1) 1L pembrolizumab + axtinib; (2) 1L nivolumab + ipilimumab)) | Pembrolizumab-axitinib is associated with greater QALYs compared with nivolumab/ipilimumab but may not be cost-effective. Price reductions may make the cost of pembrolizumab-axitinib proportional to its clinical value and less financially burdensome to the US health care system. |
| [95] | 2020 | DES | HO  (QALE) | Bladder  (BCG‐unresponsive non‐muscle invasive) | HIC | R (simmer package) | None | (1) early radical cystectomy; (2) novel therapies with varying levels of effectiveness | Current recommendations regarding clinically meaningful outcomes for single-arm trials evaluating the efficacy of novel therapies may be too low and these thresholds may need to be increased to at least 45%-55% at 6 months and 35% at 18-24 months (complete response rates/recurrence-free survival). |
| [96] | 2020 | STMS | CE | Lung  (medically operable stage I NSCLC) | HIC | C++ (Microsoft Excel Professional Plus, IBM SPSS) | DSA & PSA | (1) SBRT; (2) VAST resection | SBRT dominates VATS resection in the majority of simulations. |
| [97] | 2021 | DES | CE | Multiple myeloma (transplant-ineligible, elderly) | HIC | UnS | None | 30 treatment sequences including up to 3 lines of therapy (agents used: Bortezomib, Carfilzomib, Daratumumab, Dexamethasone, Elotuzumab, Lenalidomide, Melphalan, Panobinostat, Pomalidomide, Prednisone, Thalidomide) | Sequences including novel treatments were highly effective, but the CE ratios were above currently accepted WTP thresholds. |
| [98] | 2021 | STMS | CE | Colorectal (microsatellite-instability-high/mismatch repair-deficient advanced) | HIC | R | DSA & PSA | (1) 1L pembrolizumab; (2) 2L pembrolizumab; (3) No pembrolizumab | Pembrolizumab for 2L line use is dominated by its 1L use, and 1L use is cost-effective compared with chemotherapy. |
| [99] | 2021 | STMS | CE | Colorectal (advanced rectal with complete clinical response after neoadjuvant chemoradiation) | HIC | TreeAge Pro | DSA & PSA | (1) Watch and wait; (2) Abdominoperineal resection; (3) Low anterior resection | Watch and wait is dominant. |
| [100] | 2021 | DES | CE | Prostate  (favorable-risk localized) | HIC | R | None | (1) AS; (2) Radical Prostatectomy; (3) Radiotherapy | Radiotherapy is dominant. |
| [101] | 2021 | STMS | CE | Ovarian  (advanced) | HIC | TreeAge Pro | DSA & PSA | (1) Standard evaluation; (2) Laparoscopy | Laparoscopy is a cost-effective way to improve primary treatment planning. |
| [102] | 2021 | STMS | HO  (QALE) | Bladder  (upper tract urothelial carcinoma) | UnS | TreeAge Pro | DSA | (1) Nephroureterectomy; (2) Neoadjuvant chemotherapy (before nephroureterectomy); (3) Adjuvant chemotherapy (after) | Neoadjuvant chemotherapy provides the longest QALE. |
| [103] | 2021 | STMS | HO  (QALYs) | Hodgkin lymphoma  (stage I/IIa) | HIC | UnS | None | (1) Chemotherapy, (2) chemotherapy + radiotherapy | Consolidative radiotherapy was more favorable the younger the patient, when future health discounting was included, and in never smokers. |
| [104] | 2021 | STMS | CE | Prostate  (low risk) | HIC | TreeAge Pro | DSA & PSA | (1) PSA every 6 months & requisite annual biopsy, no MRI; (2) PSA & annual MRI, annual biopsy regardless of MRI results; (3) No MRI if PSA is stable, otherwise annual MRI by using PI-RADS of ≥3 to determine need for biopsy; (4) MRI on PRIAS schedule (at years 1, 3, 7, 10, & then every 5 years) by using PI-RADS ≥4 to determine need for biopsy; (5) annual MRI by using PI-RADS ≥4 to determine need for biopsy; (6) annual MRI by using PI-RADS ≥3 to determine need for biopsy | AS with biopsy decisions guided by findings from annual MRI reduces the number of biopsies while preserving LE and quality of life. Biopsy in lesions with PI-RADS scores ≥4 is likely the most cost-effective AS strategy for men <70 years. |
| [105] | 2021 | STMS | CE | Prostate  (low-volume metastatic) | HIC | TreeAge Pro | DSA & PSA | (1) ADT + radiotherapy; (2) ADT | ADT + radiotherapy is dominant. |
| [106] | 2021 | STMS | CE | Kidney  (advanced renal cell carcinoma) | HIC | TreeAge Pro | DSA & PSA | (1) Lenvatinib + pembrolizumab: (2) Nivolumab + cabozantinib; (3) Nivolumab + ipilimumab; (4) Pembrolizumab + axitinib; (5) Avelumab + axitinib; (6) Sunitinib | Pembrolizumab + axitinib is likely to be the most cost-effective alternative at the WTP threshold of $100,000. |
| [107] | 2021 | STMS | CE | Lung  (untreated advanced anaplastic lymphoma kinase-positive NSCLC) | HIC | TreeAge Pro | DSA & PSA | (1) 1L lorlatinib; (2) 1L crizotinib | Lorlatinib was unlikely to be cost effective compared with crizotinib at a WTP threshold of 200,000/QALY. |
| [108] | 2021 | STMS | CE | Kidney  (advanced renal cell carcinoma) | HIC | TreeAge Pro | DSA & PSA | (1) 1L nivolumab + cabozantinib; (2) 1L sunitinib | Substituting nivolumab + cabozantinib in the 1L setting is unlikely to be cost-effective under the current WTP threshold ($150,000/QALY). |
| [109] | 2021 | STMS | HO  (QALE) | Bladder  (muscle-invasive) | UnS | TreeAge Pro | DSA | (1) Radical cystectomy; (2) Trimodal therapy | Radical cystectomy results in a longer life expectancy compared to trimodal therapy (0.54 years), but a lower QALE (-0.07 years). The preferred treatment strategy varied with patient age. |
| [110] | 2021 | STMS | HO (progression free & overall survival) | Multiple myeloma | UnS | R | None | (1) Carfilzomib + lenalidomide + dexamethasone; (2) Lenalidomide + dexamethasone | The model had a good fit with the ASPIRE trial data. The addition of carfilzomib improves progression-free survival. |
| [111] | 2021 | STMS | CE | Liver  (locally advanced hepatocellular carcinoma) | HIC | TreeAge Pro, R | DSA | (1) SIRT; (2) Sorafenib | Sorafenib is unlikely to provide a gain in quality-adjusted survival compared with SIRT at an acceptable cost for the US healthcare sector. |
| [112] | 2021 | STMS | CE | Skin  (BRAF-mutant resected stage III melanoma) | HIC | TreeAge Pro | DSA & PSA | (1) Ipilimumab; (2) Nivolumab; (3) Pembrolizumab: (4) Dabrafenib + trametinib; (5) No adjuvant treatment | Pembrolizumab is cost-effective at a conventional WTP threshold, dabrafenib–trametinib is not. |
| [113] | 2021 | STMS | CE | Breast  (low-risk DCIS) | HIC | TreeAge Pro, R | DSA & PSA | (1) APBI with IMRT; (2) APBI with IMRT + AI; (3) No adjuvant therapy | No adjuvant therapy is most cost-effective for postmenopausal women 60 years or older who receive partial mastectomy for low-risk DCIS. |
| [114] | 2021 | STMS | HO  (5-year net survival) | Breast | UnS | Java | None | Scaling up imaging (ultrasound, x-ray, CT, MRI, PET, SPECT), treatment (chemotherapy, radiotherapy, surgery, targeted therapy) & quality of care to mean level of HIC, individually and in combination. | Scale-up of treatment and imaging modalities, and improvements in quality of care could improve global 5-year net survival by nearly 15 percentage points. Scale-up of traditional modalities and quality-of-care improvements could achieve 70% of these total potential gains, with substantial impact in LMIC, providing a more feasible pathway to improving survival in these settings even without the benefits of future investments in targeted therapy and advanced imaging. |
| [115] | 2022 | STMS | CE | CNS  (pediatric low-grade glioma) | HIC | UnS | None | (1) Molecular testing for BRAF fusion; (2) No molecular testing | Molecular testing is cost-effective. |

**1L**: first-line; **2L**: second-line; **3L**: third-line;**3DCRT**: 3D conformal radiotherapy; **AI**: aromatase inhibitor; **ADT**: androgen deprivation therapy; **APBI**: accelerated partial breast irradiation; **AS**: active surveillance; **BRCA**: breast cancer gene; **CAR-T**: chimeric antigen t-cell; **CE**: cost-effectiveness; **CHOP**: cyclophosphamide, hydroxydaunorubicin, oncovin, prednisone; **CHOP-R**: CHOP, rituximab; **CNS**: central nervous system; **CO**: cost outcomes; **CT**: computerized tomography; **CVP-R**: cyclofosfamide, vincristine, prednison rituximab; **DCIS**: ductal carcinoma in situ; **DES**: discrete event simulation; **DLBCL**: diffuse large B-cell lymphoma; **DSA:** deterministic sensitivity analysis; **HIC**: high-income country; **HO**: health outcomes; **HRD**: homologous recombination deficiency; **ICER**: incremental cost-effectiveness ratio; **IMRT**: Intensity modulated radiation therapy; **LE**: life expectancy; **LMIC**: low- and middle income countries; **LY**: life years; **MRI**: magnetic resonance imaging; **NHL**: non-hodgkin's lymphoma; **NSCLC**: non-small cell lung cancer; **PET**: positron emission tomography; **PD-1**: programmed cell death protein 1; **PD-L1**: programmed cell death ligand 1; **PI-RADS**: Prostate Imaging Reporting and Data System; **PRIAS**: Prostate Cancer Research International Active Surveillance; **PSA**: prostate-specific antigen; **PSA (sensitivity analysis):** probabilistic sensitivity analysis;  **QALE**: quality-adjusted life expectancy; **QALY**: quality-adjusted life years; **SBRT**: stereotactic body radiotherapy; **SPECT**: single-photon emission computerized tomography; **STMS**: state-transition microsimulation; **TA**: timed automata; **TKI**: tyrosine kinase inhibitors; **UnS**: unspecified; **VATS**: video assisted thoracic surgery; **VLNT**: vascularized lymph node transfer; **WBC**: World Bank Classification; **WTP**: willingness-to-pay

**Table A2: HSCO Models**

| Paper | Year | Model | Category | Outcomes | Setting | Cancer | WBC | Software | Sensitivity analysis | Therapy | Results |
| --- | --- | --- | --- | --- | --- | --- | --- | --- | --- | --- | --- |
| [116] | 2010 | DES | Operational changes | Waiting time | Micro | UnS | HIC | UnS | None | Radiotherapy | Pooling urgent and regular patients is not always beneficial with regard to waiting times of urgent patients. Separation of queues may require less capacity to meet waiting time targets for urgent and regular patients. |
| [117] | 2011 | DES | Operational changes | Waiting time, working time, resource utilization | Micro | UnS | HIC | Rockwell Arena | DSA | Proton radiotherapy | Modeling and simulation of the irradiation process of the PROSCAN facility at the Paul Scherrer Institut. |
| [118] | 2012 | DES | Capacity planning and management | Waiting time, throughput time | Micro | UnS | HIC | MedModel simulation software v.7, ProModel Corporation. | None | Radiotherapy | Waiting times were improved by reducing the fluctuations in the outpatient department capacity. |
| [119] | 2013 | DES | Resource allocation | Throughput | Micro | Skin | HIC | Rockwell Arena | None | Photo dynamic therapy, surgery | By managing 3 factors (i.e., the admission rule, resources allocation and capacity planning) in a dermato-oncology unit throughput times for treatments of new patients can be decreased with more than 90%, even with the same resource level. |
| [120] | 2013 | DES | Patient scheduling | Waiting time | Micro | UnS | HIC | Rockwell Arena | None | Chemotherapy | The model identified nurse unavailability during oncology treatment as a bottleneck in patient flow and solutions were sought by optimizing nurse scheduling and starting time. Duke Cancer Institute implemented the following strategies to optimize its staffing: (1) hiring 5 part-time nurses to assist in meeting variable day-to-day peak demand, (2) adjusting start times for these new hires and some existing nurses to the half-hour mark (3) hiring 1.75 additional full-time equivalent nurses and (4) stopping the practice of allowing nurses to work longer shifts. |
| [121] | 2014 | STMS | Capacity planning and management | Resource utilization | Macro | Lung | HIC | Python | None | Surgery | At the current rate of training, incidence of operable lung cancer will increase until 2030 and then plateau and decline. The increase will outstrip supply of thoracic surgeons, but the decline after 2030 will translate into an excess future supply. Minor increases in rate of training in response to short-term needs could be problematic in the longer term. Unregulated workforce changes should therefore be approached with care. |
| [122] | 2015 | DES | Patient scheduling | Waiting time, working time | Micro | UnS | HIC | Anylogic | None | Chemotherapy | Patient waiting times and clinic total working times can be reduced and a more balanced resource utilization can be achieved by using better scheduling methods. |
| [123] | 2016 | DES | Patient scheduling | Waiting time, working time, resource utilization | Micro | UnS | HIC | Rockwell Arena | None | Chemotherapy | The hematology-oncology clinic in Québec experienced a 20% increase for hematology treatments and a 131% increase for oncology treatments. A nurse overload problem is observed with a nurse occupancy rate of 86.98% in the morning and 64.48%in the afternoon. New schedule appointments taking into account nurse capacity result in a decrease of the difference in nurse occupancy rates in the morning and in the afternoon. |
| [124] | 2016 | STMS | Capacity planning and management | Resource utilization | Macro | Lung | HIC | Python | None | SBRT | With the implementation of SBRT for treatment of early-stage NSCLC, there would be a decrease in operative volume. The impact would depend on the stage of NSCLC for which SBRT is recommended and on compliance. A national strategy for thoracic surgery workforce planning is necessary, given the complex interaction of CT screening and the treatment of medically operable early NSCLC with SBRT. |
| [125] | 2017 | DES | Capacity planning and management | Waiting time | Micro | UnS | HIC | Simul8 | None | Radiotherapy | The radiotherapy planning process at the London Regional Cancer Program was analysed to determine the bottlenecks and to quantify the effect of specific resource levels with the goal of reducing waiting times. |
| [126] | 2017 | DES | Misc. | Waiting time | Micro | UnS | HIC | Rockwell Arena | None | UnS | Incorporating both nurses’ direct as indirect tasks in a haematology-oncology clinic results in more realistic DES model. |
| [127] | 2017 | DES | Patient scheduling | Overtime | Micro | Colorectal | HIC | UnS | None | Surgery | A methodological framework to make efficient use of scarce resources with a goal of coordinating clinic and surgery appointments so that patients with different acuity levels can see a surgeon in the clinic and schedule their surgery within a maximum wait time target that is clinically safe for them. Heuristic scheduling policies are proposed with 2 underlying ideas behind them: (1) proactively book a tentative surgery day along with the clinic appointment at the time an appointment request is received, and (2) intelligently space out clinic and surgery appointments such that if the patient does not need his/her surgery appointment there is sufficient time to offer it to another patient. |
| [128] | 2017 | DES | Operational changes | Throughput | Micro | UnS | LMIC | MATLAB | None | UnS | An early discharge approach can reduce the number of turned away patients by 10% in the Kidney Department, equivalent to 182 patients annually and by 11% in the Oncology Department, equivalent to 150 patients annually. |
| [129] | 2018 | DES | Patient scheduling | Overtime, throughput time, throughput, resource utilization | Micro | UnS | HIC | DEVSJAVA | None | Chemotherapy | The model analysed how patients and clinic resources should be scheduled and how the number of nurses impacts clinic performance. |
| [130] | 2018 | DES | Operational changes | Throughput time | Micro | UnS | LMIC | ProModel 6.0 | None | Chemotherapy, radiotherapy | Six Sigma process improvement methodology identified fragmented and unstandardized processes and procedures and a lack of communication among the stakeholders as leading causes of long discharge times. Categorizing patients by their needs enabled better design of the discharge processes. Simplified and standardized processes, improved communications, and system-wide management and other proposed improvements reduced patient discharge time by 54% from 216 minutes. |
| [131] | 2018 | DES | Operational changes | Waiting time | Micro | Breast | LMIC | Rockwell Arena | None | Surgery | A DES model was developed to identify bottlenecks in patient flow and 4 scenario's to decrease waiting time were analysed. The best scenario involved adding an additional breast surgeon. |
| [132] | 2018 | DES | Operational changes | Waiting time, throughput time, resource utilization, cost | Micro | UnS | HIC | Rockwell Arena | None | UnS | Although a high level Health Information System lengthens the consultation, occupation rate of oncologists are lower and quality of service is higher (through the number of available information accessed during consultation to formulate the diagnostic). The model can determine the most cost-effective ICT elements to improve the care process quality while minimizing costs. |
| [133] | 2019 | DES | Operational changes | Throughput time | Micro | Bladder | HIC | Simul8 | None | Surgery, chemotherapy | Cost-neutral practical changes to the pathway led to significant reductions in delays for bladder cancer patients at Royal Cornwall Hospital. |
| [134] | 2019 | DES | Operational changes | Waiting time, throughput time | Micro | Pediatric | HIC | Rockwell Arena | None | Antibiotics | A time-specific protocol designed to decrease time to antibiotic administration for children with cancer and central venous catheters presenting to the paediatric ED with fever has no significant impact on patient flow. The model suggests system resilience, demonstrating no detrimental effect on WT until there is a 7- fold increase in the proportion of patients receiving the protocol. |
| [135] | 2019 | DES | Patient scheduling | Waiting time | Micro | Multiple | HIC | Tecnomatix Siemens Plant Simulation 13.2 by Siemens PLM Software | None | Radiotherapy | A 100% pull strategy (i.e., first treatment is set after consultation) allows for more patients starting treatment within the waiting time targets than a hybrid strategy (i.e., combination of pull and push where push refers to a strategy in which the first treatment is set after completion pretreatment workflow), in spite of slightly longer waiting times and more first appointment rebooks. |
| [136] | 2020 | DES | Resource allocation | Working time, resource utilization | Micro | UnS | HIC | Rockwell Arena | DSA | Chemotherapy | Not all chemotherapy protocols result in similar workloads. Both physical and mental nurse workload should be taken into account in determining the nurse-patient ratio for the administration of chemotherapy treatment. |
| [137] | 2020 | DES | Resource scheduling | Waiting time, throughput time | Micro | UnS | HIC | FlexSim Healthcare | None | Chemotherapy | New nursing schedules for the infusion centre and improved pharmacy processes have positive impacts on reducing patient waiting times by approximately 20% and overall length of stay by approximately 3.4% to 4.6%. |
| [138] | 2020 | DES | Capacity planning and management | Waiting time, costs | Micro | UnS | HIC | Rockwell Arena | None | Chemotherapy | By adding one nurse dedicated to contacting patients prior to their chemotherapy, the hospital could increase its revenue while reducing waiting times. |
| [139] | 2021 | DES | Misc. | Health outcomes | Micro | Multiple | HIC | R (simmer package) | DSA & PSA | Chemotherapy, radiotherapy, surgery | Pandemic related delays of >2 months for cancer treatment are estimated in 6–8% of patients. Additional cancer deaths at 5 years will be observed for 2020 patients and the impact is mainly found on sarcoma, gynaecological, liver, head and neck, breast cancer and acute leukaemia. |
| [140] | 2021 | DES | Patient scheduling | Waiting time, throughput time | Micro | Multiple | HIC | Rockwell Arena | None | Chemotherapy | A mixed integer programming model was developed that assigns starting day of treatment for new patients and finds the optimum number of needed nurses and pharmacists to fulfil two objectives. DES model is used to generate patient appointment schedules that minimise treatment delay for patients and total completion times of treatments in each day under resources availability constraints, including 2 new constraints covering the drug availability and pharmacists working-hours. |
| [141] | 2021 | DES | Patient scheduling | Waiting time, throughput | Micro | UnS | LMIC | Rockwell Arena | None | Radiotherapy | The model was able to identify better configurations of the process, improving productivity without deteriorating quality. This work demonstrates the possibility of applying modern simulation techniques in radiotherapy, not only enabling improvements for the present situation but also integrating the model to the operation to support everyday decision making. |
| [142] | 2021 | DES | Patient scheduling | Waiting time, overtime | Micro | UnS | HIC | Microsoft Excel, Visual Basic for Applications | None | Chemotherapy | A schedule that assigns patients to 2-3 different appointment times based on the expected length of their chemotherapy infusion can reduce average patient waiting time and nurse overtime. |
| [143] | 2022 | DES | Patient scheduling | Waiting time, overtime, throughput | Micro | Skin | HIC | Rockwell Arena | None | Mohs Micrographic Surgery | The number of patients scheduled changes the ideal spacing between appointments. Mohs Micrographic Surgery clinics can benefit from using this simulation model to explore new scheduling templates, especially when reduced patient waiting time and clinic overtime is a priority. |
| [144] | 2022 | ABM | Operational changes | Waiting time | Micro | UnS | HIC | Netlogo | None | Chemotherapy | A fixed batch size with a low number of therapies and an effective appointment strategy significantly decrease the patient waiting time in an oncology department. |

**ABM:** agent-based model; **CT**: computed tomography; **DES**: discrete event simulation; **DSA:** deterministic sensitivity analysis; **HIC**: high-income country; **LMIC**: low- and middle income countries; **NSCLC**: non-small-cell lung cancer; **PSA:** probabilistic sensitivity analysis; **SBRT**: stereotactic ablative radiotherapy; **STMS**: state-transition microsimulation**; UnS**: unspecified; **WBC**: World Bank Classification

1. Siebert, U., et al., *State-transition modeling: a report of the ISPOR-SMDM modeling good research practices task force–3.* Medical Decision Making, 2012. **32**(5): p. 690-700.

2. Krijkamp, E.M., et al., *Microsimulation Modeling for Health Decision Sciences Using R: A Tutorial.* Medical Decision Making, 2018. **38**(3): p. 400-422.

3. Zucchelli, E., A. Jones, and N. Rice, *The evaluation of health policies through microsimulation methods*. 2010, HEDG, c/o Department of Economics, University of York.

4. Bespalov, A., et al., *Cancer screening simulation models: a state of the art review.* BMC Medical Informatics and Decision Making, 2021. **21**(1): p. 359.

5. Zhang, X., *Application of discrete event simulation in health care: a systematic review.* BMC Health Serv Res, 2018. **18**(1): p. 687.

6. Vázquez-Serrano, J.I., R.E. Peimbert-García, and L.E. Cárdenas-Barrón, *Discrete-Event Simulation Modeling in Healthcare: A Comprehensive Review.* Int J Environ Res Public Health, 2021. **18**(22).

7. Karnon, J., et al., *Modeling Using Discrete Event Simulation: A Report of the ISPOR-SMDM Modeling Good Research Practices Task Force–4.* Medical Decision Making, 2012. **32**(5): p. 701-711.

8. Caro, J.J., et al., *Discrete event simulation for health technology assessment*. 2015: CRC press.

9. Caro, J.J., et al., *Modeling good research practices—overview: a report of the ISPOR-SMDM Modeling Good Research Practices Task Force–1.* Medical Decision Making, 2012. **32**(5): p. 667-677.

10. Wang, Z., et al., *Integrated PK-PD and agent-based modeling in oncology.* J Pharmacokinet Pharmacodyn, 2015. **42**(2): p. 179-89.

11. Wang, Z., et al., *Simulating cancer growth with multiscale agent-based modeling.* Semin Cancer Biol, 2015. **30**: p. 70-8.

12. Zhang, L., et al., *Multiscale agent-based cancer modeling.* Journal of Mathematical Biology, 2009. **58**(4): p. 545-559.

13. Degeling, K., et al., *Comparison of Timed Automata with Discrete Event Simulation for Modeling of Biomarker-Based Treatment Decisions: An Illustration for Metastatic Castration-Resistant Prostate Cancer.* Value in Health, 2017. **20**(10): p. 1411-1419.

14. Johnston, K.M., et al., *Cost-effectiveness of the addition of rituximab to CHOP chemotherapy in first-line treatment for diffuse large B-cell lymphoma in a population-based observational cohort in British Columbia, Canada.* Value Health, 2010. **13**(6): p. 703-11.

15. Iannazzo, S., et al., *Cost-effectiveness analysis of LHRH agonists in the treatment of metastatic prostate cancer in Italy.* Value Health, 2011. **14**(1): p. 80-9.

16. Möller, J., L. Nicklasson, and A. Murthy, *Cost-effectiveness of novel relapsed-refractory multiple myeloma therapies in Norway: lenalidomide plus dexamethasone vs bortezomib.* J Med Econ, 2011. **14**(6): p. 690-7.

17. Hummel, S.R., et al., *A model of the cost-effectiveness of intensity-modulated radiotherapy in comparison with three-dimensional conformal radiotherapy for the treatment of localised prostate cancer.* Clin Oncol (R Coll Radiol), 2012. **24**(10): p. e159-67.

18. Kymes, S.M., et al., *Economic evaluation of plerixafor for stem cell mobilization.* Am J Manag Care, 2012. **18**(1): p. 33-41.

19. Evans, W.K., et al., *CANADIAN CANCER RISK MANAGEMENT MODEL: EVALUATION OF CANCER CONTROL.* International Journal of Technology Assessment in Health Care, 2013. **29**(2): p. 131-139.

20. Fragoulakis, V., et al., *Economic evaluation of therapies for patients suffering from relapsed-refractory multiple myeloma in Greece.* Cancer Management and Research, 2013. **5**(1): p. 37-48.

21. Johnston, C.F., et al., *The management of patients with T1 adenocarcinoma of the low rectum: a decision analysis.* Dis Colon Rectum, 2013. **56**(4): p. 400-7.

22. Leunis, A., et al., *The development and validation of a decision-analytic model representing the full disease course of acute myeloid leukemia.* PharmacoEconomics, 2013. **31**(7): p. 605-621.

23. Soeteman, D.I., et al., *Modeling the Effectiveness of Initial Management Strategies for Ductal Carcinoma In Situ.* JNCI: Journal of the National Cancer Institute, 2013. **105**(11): p. 774-781.

24. Tappenden, P., et al., *Using whole disease modeling to inform resource allocation decisions: economic evaluation of a clinical guideline for colorectal cancer using a single model.* Value Health, 2013. **16**(4): p. 542-53.

25. Tramontano, A.C., et al., *Microsimulation Model Predicts Survival Benefit of Radiofrequency Ablation and Stereotactic Body Radiotherapy Versus Radiotherapy for Treating Inoperable Stage I Non-Small Cell Lung Cancer.* American Journal of Roentgenology, 2013. **200**(5): p. 1020-1027.

26. Dewilde, S., et al., *Bendamustine-rituximab: a cost-utility analysis in first-line treatment of indolent non-Hodgkin's lymphoma in England and Wales.* J Med Econ, 2014. **17**(2): p. 111-24.

27. Dragomir, A., et al., *Drug costs in the management of metastatic castration-resistant prostate cancer in Canada.* Bmc Health Services Research, 2014. **14**.

28. Graczyk, J., et al., *Granulocyte colony-stimulating factor as secondary prophylaxis of febrile neutropenia in the management of advanced-stage Hodgkin lymphoma treated with adriamycin, bleomycin, vinblastine and dacarbazine chemotherapy: a decision analysis.* Leuk Lymphoma, 2014. **55**(1): p. 56-62.

29. Hoyer, T., et al., *Cost-Effectiveness of Early-Initiated Treatment for Advanced-Stage Epithelial Ovarian Cancer Patients A Modeling Study.* International Journal of Gynecological Cancer, 2014. **24**(1): p. 75-84.

30. Kongnakorn, T., et al., *Economic implications of using bendamustine, alemtuzumab, or chlorambucil as a first-line therapy for chronic lymphocytic leukemia in the US: A cost-effectiveness analysis.* ClinicoEconomics and Outcomes Research, 2014. **6**(1): p. 141-149.

31. Louie, A.V., et al., *Measuring the population impact of introducing stereotactic ablative radiotherapy for stage I non-small cell lung cancer in Canada.* Oncologist, 2014. **19**(8): p. 880-5.

32. Blakely, T., et al., *Cancer care coordinators in stage III colon cancer: a cost-utility analysis.* BMC Health Serv Res, 2015. **15**: p. 306.

33. Jahn, B., et al., *Cost effectiveness of personalized treatment in women with early breast cancer: the application of OncotypeDX and Adjuvant! Online to guide adjuvant chemotherapy in Austria.* Springerplus, 2015. **4**.

34. Savitz, S.T., R.C. Chen, and D.J. Sher, *Cost-effectiveness analysis of neurocognitive-sparing treatments for brain metastases.* Cancer, 2015. **121**(23): p. 4231-9.

35. Arrieta, O., et al., *Cost-effectiveness analysis of EGFR mutation testing in patients with non-small cell lung cancer (NSCLC) with gefitinib or carboplatin–paclitaxel.* European Journal of Health Economics, 2016. **17**(7): p. 855-863.

36. De Groot, S., et al., *Potential health gains for patients with metastatic renal cell carcinoma in daily clinical practice: A real-world cost-effectiveness analysis of sequential first- and second-line treatments.* PLoS One, 2017. **12**(5): p. e0177364.

37. Borg, S., et al., *Cost effectiveness of pomalidomide in patients with relapsed and refractory multiple myeloma in Sweden.* Acta Oncol, 2016. **55**(5): p. 554-60.

38. Collinson, L., et al., *Economic evaluation of single-fraction versus multiple-fraction palliative radiotherapy for painful bone metastases in breast, lung and prostate cancer.* J Med Imaging Radiat Oncol, 2016. **60**(5): p. 650-660.

39. Davies, K.R., et al., *Outcomes of contralateral prophylactic mastectomy in relation to familial history: a decision analysis (BRCR-D-16-00033).* Breast Cancer Res, 2016. **18**(1): p. 93.

40. *Minimal Residual Disease Evaluation in Childhood Acute Lymphoblastic Leukemia: An Economic Analysis.* Ont Health Technol Assess Ser, 2016. **16**(8): p. 1-83.

41. Jahn, B., et al., *Lessons Learned from a Cross-Model Validation between a Discrete Event Simulation Model and a Cohort State-Transition Model for Personalized Breast Cancer Treatment.* Med Decis Making, 2016. **36**(3): p. 375-90.

42. Quik, E.H., et al., *Individual patient information to select patients for different radiation techniques.* European Journal of Cancer, 2016. **62**: p. 18-27.

43. Shrime, M.G., et al., *Time-Limited Trials of Intensive Care for Critically Ill Patients With Cancer: How Long Is Long Enough?* JAMA Oncol, 2016. **2**(1): p. 76-83.

44. Stenehjem, D.D., et al., *Cost-Utility of a Prognostic Test Guiding Adjuvant Chemotherapy Decisions in Early-Stage Non-Small Cell Lung Cancer.* Oncologist, 2016. **21**(2): p. 196-204.

45. de Carvalho, T.M., E.A.M. Heijnsdijk, and H.J. de Koning, *Estimating the risks and benefits of active surveillance protocols for prostate cancer: a microsimulation study.* Bju International, 2017. **119**(4): p. 560-566.

46. de Carvalho, T.M., E.A.M. Heijnsdijk, and H.J. de Koning, *When should active surveillance for prostate cancer stop if no progression is detected?* Prostate, 2017. **77**(9): p. 962-969.

47. Hörster, L., et al., *Cost-effectiveness of methods in personalized medicine. Results of a decision-analytic model in patients with acute myeloid leukemia with normal karyotype.* Leuk Res, 2017. **62**: p. 84-90.

48. Jahn, B., et al., *Personalized treatment of women with early breast cancer: a risk-group specific cost-effectiveness analysis of adjuvant chemotherapy accounting for companion prognostic tests OncotypeDX and Adjuvant!Online.* BMC Cancer, 2017. **17**(1): p. 685.

49. Mamiya, H., et al., *Cost-effectiveness of palbociclib in hormone receptor-positive advanced breast cancer.* Ann Oncol, 2017. **28**(8): p. 1825-1831.

50. Parikh, R.C., et al., *Cost-Effectiveness of Treatment Sequences of Chemotherapies and Targeted Biologics for Elderly Metastatic Colorectal Cancer Patients.* Journal of Managed Care & Specialty Pharmacy, 2017. **23**(1): p. 64-73.

51. Raphael, J., et al., *Palbociclib in hormone receptor positive advanced breast cancer: A cost-utility analysis.* Eur J Cancer, 2017. **85**: p. 146-154.

52. Rodin, D., et al., *Cost-Effectiveness Analysis of Radiation Therapy Versus Transoral Robotic Surgery for Oropharyngeal Squamous Cell Carcinoma.* Int J Radiat Oncol Biol Phys, 2017. **97**(4): p. 709-717.

53. Wang, H.I., et al., *Treatment cost and life expectancy of diffuse large B-cell lymphoma (DLBCL): a discrete event simulation model on a UK population-based observational cohort.* Eur J Health Econ, 2017. **18**(2): p. 255-267.

54. Birnbaum, J.K., et al., *Early detection and treatment strategies for breast cancer in low-income and upper middle-income countries: a modelling study.* Lancet Glob Health, 2018. **6**(8): p. e885-e893.

55. Chen, Q., et al., *Exploring the potential cost-effectiveness of precision medicine treatment strategies for diffuse large B-cell lymphoma.* Leuk Lymphoma, 2018. **59**(7): p. 1700-1709.

56. Degeling, K., et al., *Matching the model with the evidence: comparing discrete event simulation and state-transition modeling for time-to-event predictions in a cost-effectiveness analysis of treatment in metastatic colorectal cancer patients.* Cancer Epidemiology, 2018. **57**: p. 60-67.

57. Pan, F., et al., *Modeling Clinical Outcomes in Prostate Cancer: Application and Validation of the Discrete Event Simulation Approach.* Value in Health, 2018. **21**(4): p. 416-422.

58. Tarhini, A., et al., *Sequential treatment approaches in  the management of BRAF wild-type advanced melanoma: a cost-effectiveness analysis.* Immunotherapy, 2018. **10**(14): p. 1241-1252.

59. Wang, H.I., et al., *A Generic Model for Follicular Lymphoma: Predicting Cost, Life Expectancy, and Quality-Adjusted-Life-Year Using UK Population–Based Observational Data.* Value in Health, 2018. **21**(10): p. 1176-1185.

60. Weaver, D.T., et al., *Modeling treatment outcomes for patients with advanced ovarian cancer: Projected benefits of a test to optimize treatment selection.* Gynecol Oncol, 2018. **149**(2): p. 256-262.

61. Abraham, P., et al., *Cost-effectiveness of intraoperative MRI for treatment of high-grade gliomas.* Radiology, 2019. **291**(3): p. 689-697.

62. Criss, S.D., et al., *Cost-effectiveness and Budgetary Consequence Analysis of Durvalumab Consolidation Therapy vs No Consolidation Therapy After Chemoradiotherapy in Stage III Non-Small Cell Lung Cancer in the Context of the US Health Care System.* JAMA Oncol, 2019. **5**(3): p. 358-365.

63. Criss, S.D., et al., *Cost-effectiveness of Atezolizumab Combination Therapy for First-Line Treatment of Metastatic Nonsquamous Non-Small Cell Lung Cancer in the United States.* JAMA Netw Open, 2019. **2**(9): p. e1911952.

64. Criss, S.D., et al., *Effect of PD-L1 testing on the cost-effectiveness and budget impact of pembrolizumab for advanced urothelial carcinoma of the bladder in the United States.* Urol Oncol, 2019. **37**(3): p. 180.e11-180.e18.

65. Nhut, D.T. and B. Jan, *Adjuvant trastuzumab chemotherapy in early breast cancer: meta-analysis of randomised trials and cost-effectiveness analysis.* Swiss Medical Weekly, 2019. **149**.

66. Fernandes, R.R.A., et al., *Cost-Effectiveness of Proton Versus Photon Therapy in Pediatric Medulloblastoma Treatment: A Patient Volume–Based Analysis.* Value in Health Regional Issues, 2019. **20**: p. 122-128.

67. Gibson, E.J., et al., *Cohort versus patient level simulation for the economic evaluation of single versus combination immuno-oncology therapies in metastatic melanoma.* J Med Econ, 2019. **22**(6): p. 531-544.

68. Hajjar, A., et al., *Cost-effectiveness of adjuvant paclitaxel and trastuzumab for early-stage node-negative, HER2-positive breast cancer.* PLoS One, 2019. **14**(6): p. e0217778.

69. Kim, K., et al., *Cost effectiveness of therapeutic drug monitoring for imatinib administration in chronic myeloid leukemia.* PLoS One, 2019. **14**(12): p. e0226552.

70. Lietz, A.P., et al., *Potential survival benefits from optimized chemotherapy implementation in advanced ovarian cancer: Projections from a microsimulation model.* PLoS One, 2019. **14**(9): p. e0222828.

71. Sarkar, R.R., et al., *Cost-Effectiveness of Chimeric Antigen Receptor T-Cell Therapy in Pediatric Relapsed/Refractory B-Cell Acute Lymphoblastic Leukemia.* J Natl Cancer Inst, 2019. **111**(7): p. 719-726.

72. Sexton Ward, A., et al., *The long-term social value of granulocyte colony-stimulating factors.* Am J Manag Care, 2019. **25**(10): p. 486-493.

73. Tarhini, A., et al., *Clinical and economic outcomes associated with treatment sequences in patients with BRAF-mutant advanced melanoma.* Immunotherapy, 2019. **11**(4): p. 283-295.

74. Tully, S., et al., *Impact of Increasing Wait Times on Overall Mortality of Chimeric Antigen Receptor T-Cell Therapy in Large B-Cell Lymphoma: A Discrete Event Simulation Model.* JCO Clin Cancer Inform, 2019. **3**: p. 1-9.

75. Wan, X., et al., *Ribociclib in hormone-receptor-positive advanced breast cancer: Establishing a value-based cost in China.* Breast, 2019. **43**: p. 1-6.

76. Ward, M.C., et al., *Radiation Therapy Without Hormone Therapy for Women Age 70 or Above with Low-Risk Early Breast Cancer: A Microsimulation.* Int J Radiat Oncol Biol Phys, 2019. **105**(2): p. 296-306.

77. Ward, Z.J., et al., *Global childhood cancer survival estimates and priority-setting: a simulation-based analysis.* Lancet Oncol, 2019. **20**(7): p. 972-983.

78. White, C., et al., *A decision analysis comparing 3 active surveillance protocols for the treatment of patients with low-risk prostate cancer.* Cancer, 2019. **125**(6): p. 952-962.

79. Ambavane, A., et al., *Clinical and economic outcomes of treatment sequences for intermediate- to poor-risk advanced renal cell carcinoma.* Immunotherapy, 2020. **12**(1): p. 37-51.

80. Bieri, U., et al., *Management of Active Surveillance-Eligible Prostate Cancer during Pretransplantation Workup of Patients with Kidney Failure: A Simulation Study.* Clin J Am Soc Nephrol, 2020. **15**(6): p. 822-829.

81. Corkum, J.P. and M. Bezuhly, *Combining vascularized lymph node transfer with autologous breast reconstruction: A Surveillance, Epidemiology and End Results (SEER) Database cost-utility analysis.* J Plast Reconstr Aesthet Surg, 2020. **73**(10): p. 1879-1888.

82. Criss, S.D., et al., *Cost-effectiveness of pembrolizumab for advanced non-small cell lung cancer patients with varying comorbidity burden.* PLoS One, 2020. **15**(1): p. e0228288.

83. Degeling, K., et al., *Simulating Progression-Free and Overall Survival for First-Line Doublet Chemotherapy With or Without Bevacizumab in Metastatic Colorectal Cancer Patients Based on Real-World Registry Data.* Pharmacoeconomics, 2020. **38**(11): p. 1263-1275.

84. Hird, A.E., et al., *Abiraterone vs. docetaxel for metastatic hormone-sensitive prostate cancer: A microsimulation model.* Cuaj-Canadian Urological Association Journal, 2020. **14**(9): p. E418-E427.

85. Ibarrondo, O., et al., *Probabilistic cost-utility analysis and expected value of perfect information for the Oncotype multigenic test: a discrete event simulation model.* Gac Sanit, 2020. **34**(1): p. 61-68.

86. Lange, J.M., et al., *Prostate cancer mortality and metastasis under different biopsy frequencies in North American active surveillance cohorts.* Cancer, 2020. **126**(3): p. 583-592.

87. Legoupil, C., et al., *A microsimulation model to assess the economic impact of immunotherapy in non-small cell lung cancer.* Erj Open Research, 2020. **6**(2).

88. Moldaver, D., et al., *Development, validation and results from the impact of treatment evolution in non-small cell lung cancer (iTEN) model.* Lung Cancer, 2020. **139**: p. 185-194.

89. Penn, C.A., M.S. Wong, and C.S. Walsh, *Cost-effectiveness of Maintenance Therapy Based on Molecular Classification Following Treatment of Primary Epithelial Ovarian Cancer in the United States.* JAMA Netw Open, 2020. **3**(12): p. e2028620.

90. Schluckebier, L., et al., *Cost-effectiveness analysis comparing companion diagnostic tests for EGFR, ALK, and ROS1 versus next-generation sequencing (NGS) in advanced adenocarcinoma lung cancer patients.* BMC Cancer, 2020. **20**(1): p. 875.

91. Ward, M.C., et al., *Cost-effectiveness analysis of endocrine therapy alone versus partial-breast irradiation alone versus combined treatment for low-risk hormone-positive early-stage breast cancer in women aged 70 years or older.* Breast Cancer Res Treat, 2020. **182**(2): p. 355-365.

92. Ward, Z.J., et al., *The role and contribution of treatment and imaging modalities in global cervical cancer management: survival estimates from a simulation-based analysis.* Lancet Oncol, 2020. **21**(8): p. 1089-1098.

93. Ward, Z.J., et al., *Estimating the impact of treatment and imaging modalities on 5-year net survival of 11 cancers in 200 countries: a simulation-based analysis.* Lancet Oncol, 2020. **21**(8): p. 1077-1088.

94. Watson, T.R., et al., *Cost-effectiveness of Pembrolizumab Plus Axitinib Vs Nivolumab Plus Ipilimumab as First-Line Treatment of Advanced Renal Cell Carcinoma in the US.* JAMA Netw Open, 2020. **3**(10): p. e2016144.

95. Wettstein, M.S., et al., *Required efficacy for novel therapies in BCG-unresponsive non-muscle invasive bladder cancer: Do current recommendations really reflect clinically meaningful outcomes?* Cancer Med, 2020. **9**(10): p. 3287-3296.

96. Wolff, H.B., et al., *Cost-effectiveness of stereotactic body radiation therapy versus video assisted thoracic surgery in medically operable stage I non-small cell lung cancer: A modeling study.* Lung Cancer, 2020. **141**: p. 89-96.

97. Blommestein, H.M., et al., *Cost-effectiveness of Novel Treatment Sequences for Transplant-Ineligible Patients With Multiple Myeloma.* JAMA Netw Open, 2021. **4**(3): p. e213497.

98. Tan, C.Q., et al., *Cost-Effectiveness of First-Line Versus Second-Line Pembrolizumab or Chemotherapy in Patients With Microsatellite-Instability-High/Mismatch Repair-Deficient Advanced Colorectal Cancer.* Frontiers in Pharmacology, 2021. **12**.

99. Cui, C.L., et al., *Cost Effectiveness of Watch and Wait Versus Resection in Rectal Cancer Patients with Complete Clinical Response to Neoadjuvant Chemoradiation.* Annals of Surgical Oncology, 2021.

100. Degeling, K., et al., *Lifetime Health and Economic Outcomes of Active Surveillance, Radical Prostatectomy, and Radiotherapy for Favorable-Risk Localized Prostate Cancer.* Value Health, 2021. **24**(12): p. 1737-1745.

101. Harrison, R.F., et al., *Cost-effectiveness of laparoscopic disease assessment in patients with newly diagnosed advanced ovarian cancer*.* Gynecologic Oncology, 2021. **161**(1): p. 56-62.

102. Hird, A.E., et al., *Neoadjuvant Versus Adjuvant Chemotherapy for Upper Tract Urothelial Carcinoma: A Microsimulation Model.* Clin Genitourin Cancer, 2021. **19**(2): p. e135-e147.

103. Jones, D.A., et al., *Informing Radiotherapy Decisions in Stage I/IIa Hodgkin Lymphoma: Modelling Life Expectancy Using Radiation Dosimetry.* Blood advances, 2021.

104. Kang, S.K., et al., *Active Surveillance Strategies for Low-Grade Prostate Cancer: Comparative Benefits and Cost-effectiveness.* Radiology, 2021. **300**(3): p. 594-604.

105. Lester-Coll, N.H., et al., *Cost-effectiveness of Prostate Radiation Therapy for Men With Newly Diagnosed Low-Burden Metastatic Prostate Cancer.* JAMA Netw Open, 2021. **4**(1): p. e2033787.

106. Li, S., et al., *Cost-Effectiveness of Frontline Treatment for Advanced Renal Cell Carcinoma in the Era of Immunotherapies.* Frontiers in Pharmacology, 2021. **12**.

107. Li, S., et al., *Cost-Effectiveness of Lorlatinib as a First-Line Therapy for Untreated Advanced Anaplastic Lymphoma Kinase-Positive Non-Small Cell Lung Cancer.* Frontiers in Oncology, 2021. **11**.

108. Li, S., et al., *Cost-Effectiveness of Nivolumab Plus Cabozantinib Versus Sunitinib as a First-Line Treatment for Advanced Renal Cell Carcinoma in the United States.* Frontiers in Pharmacology, 2021. **12**.

109. Magee, D., et al., *Trimodal therapy vs. radical cystectomy for muscle-invasive bladder cancer: A Markov microsimulation model.* Canadian Urological Association Journal, 2021. **16**(4).

110. Majer, I., et al., *Estimating and Extrapolating Survival Using a State-Transition Modeling Approach: A Practical Application in Multiple Myeloma.* Value in Health, 2021.

111. Marqueen, K.E., et al., *Cost-Effectiveness Analysis of Selective Internal Radiotherapy With Yttrium-90 Versus Sorafenib in Locally Advanced Hepatocellular Carcinoma.* JCO Oncol Pract, 2021. **17**(2): p. e266-e277.

112. Mojtahed, S.A., et al., *Cost-Effectiveness Analysis of Adjuvant Therapy for BRAF-Mutant Resected Stage III Melanoma in Medicare Patients.* Ann Surg Oncol, 2021. **28**(13): p. 9039-9047.

113. Ward, M.C., et al., *Cost-Effectiveness Analysis of No Adjuvant Therapy Versus Partial Breast Irradiation Alone Versus Combined Treatment for Treatment of Low-Risk DCIS: A Microsimulation.* JCO Oncol Pract, 2021. **17**(8): p. e1055-e1074.

114. Ward, Z.J., et al., *The impact of scaling up access to treatment and imaging modalities on global disparities in breast cancer survival: a simulation-based analysis.* Lancet Oncol, 2021. **22**(9): p. 1301-1311.

115. Rios, J.D., et al., *Clinical and economic impact of molecular testing for BRAF fusion in pediatric low-grade Glioma.* Bmc Pediatrics, 2022. **22**(1).

116. Joustra, P., E. van der Sluis, and N.M. van Dijk, *To pool or not to pool in hospitals: a theoretical and practical comparison for a radiotherapy outpatient department.* Annals of Operations Research, 2010. **178**(1): p. 77-89.

117. Corazza, U., R. Filippini, and R. Setola, *Discrete event simulation of a proton therapy facility: a case study.* Comput Methods Programs Biomed, 2011. **102**(3): p. 305-16.

118. Joustra, P.E., et al., *Reduce fluctuations in capacity to improve the accessibility of radiotherapy treatment cost-effectively.* Flexible Services and Manufacturing Journal, 2012. **24**(4): p. 448-464.

119. Romero, H.L., et al., *Admission and capacity planning for the implementation of one-stop-shop in skin cancer treatment using simulation-based optimization.* Health Care Management Science, 2013. **16**(1): p. 75-86.

120. Woodall, J.C., et al., *Improving Patient Access to Chemotherapy Treatment at Duke Cancer Institute.* Interfaces, 2013. **43**(5): p. 449-461.

121. Edwards, J.P., et al., *A novel approach for the accurate prediction of thoracic surgery workforce requirements in Canada.* J Thorac Cardiovasc Surg, 2014. **148**(1): p. 7-12.

122. Liang, B.H., et al., *Improvement of chemotherapy patient flow and scheduling in an outpatient oncology clinic.* International Journal of Production Research, 2015. **53**(24): p. 7177-7190.

123. Baril, C., et al., *Studying nurse workload and patient waiting time in a hematology-oncology clinic with discrete event simulation.* IIE Transactions on Healthcare Systems Engineering, 2016. **6**(4): p. 223-234.

124. Edwards, J.P., et al., *Forecasting the impact of stereotactic ablative radiotherapy for early-stage lung cancer on the thoracic surgery workforce(aEuro).* European Journal of Cardio-Thoracic Surgery, 2016. **49**(6): p. 1599-1606.

125. Babashov, V., et al., *Reducing Patient Waiting Times for Radiation Therapy and Improving the Treatment Planning Process: a Discrete-event Simulation Model (Radiation Treatment Planning).* Clin Oncol (R Coll Radiol), 2017. **29**(6): p. 385-391.

126. Baril, C., et al., *The importance of considering resource's tasks when modeling healthcare services with discrete-event simulation: an approach using work sampling method.* Journal of Simulation, 2017. **11**(2): p. 103-114.

127. Kazemian, P., et al., *Coordinating clinic and surgery appointments to meet access service levels for elective surgery.* J Biomed Inform, 2017. **66**: p. 105-115.

128. Mohamed, I., I. El-Henawy, and R.Z. El-Din, *An early discharge approach for managing hospital capacity.* International Journal of Modeling Simulation and Scientific Computing, 2017. **8**(1).

129. Alvarado, M.M., et al., *Modeling and simulation of oncology clinic operations in discrete event system specification.* Simulation-Transactions of the Society for Modeling and Simulation International, 2018. **94**(2): p. 105-121.

130. Arafeh, M., et al., *Using Six Sigma DMAIC Methodology and Discrete Event Simulation to Reduce Patient Discharge Time in King Hussein Cancer Center.* J Healthc Eng, 2018. **2018**: p. 3832151.

131. Camgoz-Akdag, H. and D. Arsoy-Ilikan, *IMPROVEMENT OF PATIENT PATHWAY IN A BREAST CANCER CENTER.* Iioab Journal, 2018. **9**(1): p. 16-26.

132. Rejeb, O., et al., *Performance and cost evaluation of health information systems using micro-costing and discrete-event simulation.* Health Care Manag Sci, 2018. **21**(2): p. 204-223.

133. Chalk, D., et al., *Reducing delays in the diagnosis and treatment of muscle-invasive bladder cancer using simulation modelling.* Journal of Clinical Urology, 2019. **12**(2): p. 129-133.

134. McKinley, K.W., et al., *Discrete event simulation modelling to evaluate the impact of a quality improvement initiative on patient flow in a paediatric emergency department.* Emergency Medicine Journal, 2019.

135. Vieira, B., et al., *Improving workflow control in radiotherapy using discrete-event simulation.* BMC medical informatics and decision making, 2019. **19**(1): p. 199.

136. Baril, C., V. Gascon, and J. Miller, *Design of experiments and discrete-event simulation to study oncology nurse workload.* IISE Transactions on Healthcare Systems Engineering, 2020. **10**(1): p. 74-86.

137. Kang, H. and E. Haswell, *Patient Flow Analysis Using Real-Time Locating System Data: A Case Study in an Outpatient Oncology Center.* JCO Oncol Pract, 2020. **16**(12): p. e1471-e1480.

138. Lame, G., O. Jouini, and J. Stal-Le Cardinal, *Combining Soft Systems Methodology, ethnographic observation, and discrete-event simulation: A case study in cancer care.* Journal of the Operational Research Society, 2020. **71**(10): p. 1545-1562.

139. Bardet, A., et al., *Impact of COVID-19 on healthcare organisation and cancer outcomes.* Eur J Cancer, 2021. **153**: p. 123-132.

140. Heshmat, M. and A. Eltawil, *Solving operational problems in outpatient chemotherapy clinics using mathematical programming and simulation.* Annals of Operations Research, 2021. **298**(1): p. 289-306.

141. Miranda, D.M. and M.P.M. Miranda, *Discrete-event simulation applied to a radiotherapy process: a case study of a cancer center.* Brazilian Journal of Operations & Production Management, 2021. **18**(1).

142. Slocum, R.F., et al., *Improving chemotherapy infusion operations through the simulation of scheduling heuristics: a case study.* Health Systems, 2021. **10**(3): p. 163-178.

143. Burns, P., S. Konda, and M. Alvarado, *Discrete-event simulation and scheduling for Mohs micrographic surgery.* Journal of Simulation, 2022. **16**(1): p. 43-57.

144. Corsini, R.R., et al., *A configurable computer simulation model for reducing patient waiting time in oncology departments.* Health Systems.
